# Influence of Plant Fraction, Soil, and Plant Species on Microbiota: a Multikingdom Comparison

**DOI:** 10.1128/mBio.02785-19

**Published:** 2020-02-04

**Authors:** Andrzej Tkacz, Eloïne Bestion, Zhiyan Bo, Marion Hortala, Philip S. Poole

**Affiliations:** a Department of Plant Sciences, University of Oxford, Oxford, United Kingdom; CEH-Oxford

**Keywords:** microbial colonization, plant microbiota, rhizosphere, roots, phyllosphere, colonization dynamics, *Stachybotrys* infection, microbial ecology, plant-microbe interactions, rhizosphere-inhabiting microbes, soil microbiology

## Abstract

Plant roots influence the soil microbiota via physical interaction, secretion, and plant immunity. However, it is unclear whether the root fraction or soil is more important in determining the structure of the prokaryotic or eukaryotic community and whether this varies between plant species. Furthermore, the leaf (phyllosphere) and root microbiotas have a large overlap; however, it is unclear whether this results from colonization of the phyllosphere by the root microbiota. Soil, rhizosphere, rhizoplane, and root endosphere prokaryote-, eukaryote-, and fungus-specific microbiotas of four plant species were analyzed with high-throughput sequencing. The strengths of factors controlling microbiota structure were determined using permutational multivariate analysis of variance (PERMANOVA) statistics. The origin of the phyllosphere microbiota was investigated using a soil swap experiment. Global microbial kingdom analysis conducted simultaneously on multiple plants shows that cereals, legumes, and Brassicaceae establish similar prokaryotic and similar eukaryotic communities inside and on the root surface. While the bacterial microbiota is recruited from the surrounding soil, its profile is influenced by the root itself more so than by soil or plant species. However, in contrast, the fungal microbiota is most strongly influenced by soil. This was observed in two different soils and for all plant species examined. Microbiota structure is established within 2 weeks of plant growth in soil and remains stable thereafter. A reciprocal soil swap experiment shows that the phyllosphere is colonized from the soil in which the plant is grown.

## Abstract (precis)

Global microbial kingdom analysis conducted simultaneously on multiple plants shows that cereals, legumes, and Brassicaceae establish similar prokaryotic and similar eukaryotic communities inside and on the root surface. While the bacterial microbiota is recruited from the surrounding soil, its profile is influenced by the root fraction more so than by soil or plant species. However, in contrast, the fungal microbiota is most strongly influenced by soil. This was observed in two different soils and for all plant species examined, indicating conserved adaptation of microbial communities to plants. Microbiota structure is established within 2 weeks of plant growth in soil and remains stable thereafter. We observed a remarkable similarity in the structure of a plant’s phyllosphere and root microbiotas and show by reciprocal soil swap experiments that both fractions are colonized from the soil in which the plant is grown. Thus, the phyllosphere is continuously colonized by the soil microbiota.

## INTRODUCTION

In each gram of soil, there are 10^8^ to 10^10^ microbial cells (1) and probably thousands, if not millions, of different species (2). Plant roots interact with the native soil community, establishing a “plant microbiota” that inhabits both soil and plant tissues (3). The plant microbiota is normally classified into fractions, bulk soil (microorganisms living more than a few millimeters away from the plant), rhizosphere (microorganisms living within a few millimeters around the roots), rhizoplane (microorganisms tightly attached to roots), and root associated (endophytic) microorganisms. In addition, the leaf microbiota resides in the phyllosphere, while legumes also have a root nodule microbiota.

Since high-throughput sequencing became accessible, the rhizosphere, root, and leaf microbiotas of many plant species grown in different soils and under different conditions have been characterized. However, typically, only a single factor (either the microbiota fraction, soil, or the plant species) is considered at a time. For example, 30 angiosperm plants were grown in a single soil and their rhizosphere and root microbiotas screened (4). While it is interesting to observe a relative increase in Streptomyces spp. inside the roots of these plants under drought stress, we wonder either it is also true for other soils and what happens to other microbial kingdoms. Plant health depends on the bacterial community structure, as it controls soilborne fungal and oomycete communities. Using a metagenomic screen of Arabidopsis thaliana roots, the interactions between microbial kingdoms were shown to be negative. Sophisticated synthetic communities consisting of bacteria, fungi, and Oomycetes confirmed that bacteria are able to suppress the negative fungal and oomycete effects on plants (5). In order to understand the underlying community dynamics, a simplified maize-derived community was tested in its ability to suppress a plant fungal pathogen. As the 7-member community reduced fungal growth, some of its members were crucial in maintaining the community structure (6).

The importance of soils and the climate was shown using 27 inbred maize lines grown in 5 different soils over the 5-year period. By performing such a high-throughput screen, it was possible to separate a rhizosphere bacterial core species from a varied community background (7). Screening of the rice metagenome showed that the sampling fraction (bulk soil, rhizosphere, rhizoplane, and endosphere) and soil are the dominant factors shaping this plant bacterial and archaeal microbiota structure (8). A study unravelling wild-grown Brassicaceae plant leaf and root microbiota changes over plant age and genotype showed that many of the leaf-inhabiting bacteria may originate from the soil (9).

While all of these studies give us an important insight into the relative influence of plant species, plant fraction, soil, and growth conditions, we need to better understand the underlying global processes directing recruitment over time of the rhizosphere, root, and leaf prokaryotic and microbial eukaryotic microbiotas.

Here, we compare the microbiota structures of four different plant species (Arabidopsis thaliana, Medicago truncatula, Pisum sativum, and Triticum aestivum) grown under two contrasting soil conditions. We observed a remarkable convergence in the prokaryotic and eukaryotic communities of the plant-associated fractions. Diverse plants establish similar prokaryotic, eukaryotic, and fungal communities within a fraction, with the plant-associated community being distinct from that of the soil in which they are grown. This dominant effect of plant fraction over plant species or soil is only revealed when the microbiotas of fractions from multiple plant species grown in different soils are examined simultaneously.

## RESULTS

### Influence of the fraction, soil, and plant species on microbiota structure.

We examined the structures of the prokaryotic, total eukaryotic, and fungal microbiotas of four plant species grown in two different soils, nutrient-poor Bawburgh soil and nutrient-rich Wytham soil. For simplicity, we call eukaryotic community an output of 18S rRNA amplicon sequencing. The primers used to target this fragment are binding to a range of eukaryotic taxa, as follows: protists, fungi, algae, diatoms, animals, and molds. We have also run fungus-specific analysis using independent internal transcribed spacer (ITS) fragment amplification, and we refer to the output of that sequencing as “fungal community.” Hence, “eukaryotic community” contains fungi but also diverse other eukaryotic groups, while “fungal community” consists solely of fungal species and allows for their species-level annotation. Prokaryotic community is an output of broad-range 16S rRNA sequencing and contains species from both bacteria and archaea.

Microbiotas were prepared from the following different fractions: bulk soil (loose soil), rhizosphere (soil adhering to soil), rhizoplane (root biofilm removed by sonication), and root associated (ground root). In addition, the soil microbiota was characterized at the beginning of the experiment (initial soil) to capture its native community structure and throughout the experiment in potted soil without plants (unplanted soil), which were kept alongside the planted pots.

Our aim in sampling these various fractions from multiple plants grown in two soils was to determine the strongest influences on the microbiota.

**FIG 1:**
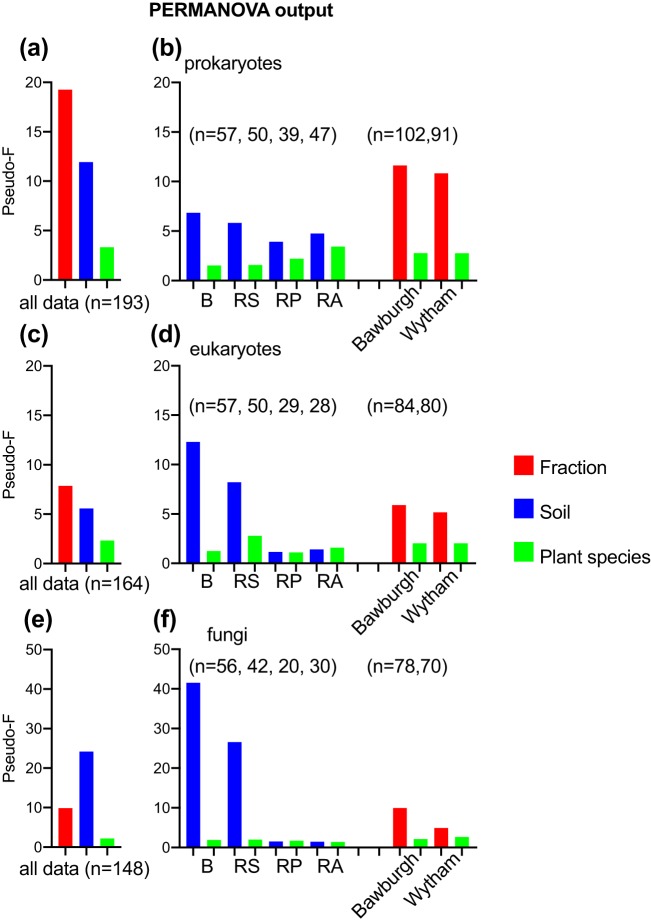
(a to f) PERMANOVA output showing importance of fraction, soil, and plant species as factors shaping the microbial community of prokaryotes (a and b), eukaryotes (c and d), and fungi (e and f). The pseudo-F value is used as a proxy for the importance of the factor. Plots represent factor importance using the all data (a, c, and e) and for specific fraction and soil (b, d, and f). B, bulk soil; RS, rhizosphere; RP, rhizoplane; RA, root associated; *n*, number of samples used in the analysis.

We ran permutational multivariate analysis of variance (PERMANOVA) using the factors fraction (bulk soil, rhizosphere, rhizoplane, and root associated), soil (Bawburgh and Wytham), and plant species (*A. thaliana*, *M. truncatula*, *P. sativum*, and *T. aestivum*) in order to establish which one of them is the strongest in shaping the prokaryotic, eukaryotic, and fungal communities. We are using the pseudo-F value as a proxy of this influence. For the prokaryotic community, the strongest influence is caused by the fraction (pseudo-F, 19.2; indicating that the variation between the groups is much larger than variations inside these groups); the next strongest influence is the soil (pseudo-F, 11.9); and the least important, yet statistically significant, influence is the plant species (pseudo-F, 3.3) (Fig. 1a). In order to test for the factor strength for individual fractions and soils, we have split the data accordingly. The soil in which the plants are grown has the strongest impact on the community in the bulk soil, while the influence was lower in the rhizosphere, rhizoplane, and root. The opposite is true for the plant species, in which influence is relatively weak in the bulk soil, with a pseudo-F value of 1.5 (yet still significant), and increases toward the root fraction, where it reaches a pseudo-F value similar to that in the soil (3.4 and 4.7 for plant species and soil, respectively). The plants exert a similar influence on their prokaryotic communities, irrespective of the soil in which they are grown. When Bawburgh- and Wytham-origin samples are analyzed separately, the fraction influence is dominant over the plant species; however, irrespective of the soil origin, they reach very similar pseudo-F values (11.6 versus 10.8 and 2.8 versus 2.8 for Bawburgh versus Wytham and fraction versus plant species, respectively) (Fig. 1b).

A rather similar data pattern can be observed for the eukaryotic community. Here, the fraction is also the strongest factor (pseudo-F, 7.8), followed by the soil (pseudo-F, 5.6) and plant species (pseudo-F, 2.3). However, the pseudo-F values and their differences between different factors are not as great as in case of the prokaryotic community (Fig. 1c). The impact of soil is especially observed for bulk soil and rhizosphere samples, while it is much smaller (yet still significant) for rhizoplane and root-associated samples. As in the case of the prokaryotic community, eukaryotic data, when split by soil into Bawburgh- and Wytham-origin samples, show a higher influence of fraction over plant species (Fig. 1d). The fungal community is very soil specific (pseudo-F, 24.2) and less dependent on fraction (pseudo-F, 9.8) or plant species (pseudo-F, 2.2) (Fig. 1e). The soil influence is very significant for bulk soil and rhizosphere fractions while weak (yet statistically significant) for the rhizoplane and root-associated communities, while the plant species influence is relatively low for all fractions. Again, as in the case of prokaryotic and eukaryotic communities, when the fungal data are split by soil, fraction is the dominant factor over plant species (Fig. 1f); however, the influence of the fraction is relatively stronger for Bawburgh than for Wytham samples. For a detailed PERMANOVA output, please refer to Table S1, sheet 1, in the supplemental material. Independent statistical tests using analysis of similarity (ANOSIM) run on the same data set indicate very similar community structure differences between fractions, soils, and plant species (Table S1, sheets 2 to 4). We have also run Canonical Analysis of Principal coordinates (CAP) analysis in order to decipher the influence of individual factors (Fig. S1). CAP shows essentially the same data trend as PERMANOVA, a clear separation of prokaryotic fractions (in both Bawburgh and Wytham soils), and a weaker (yet statistically significant) plant species influence. In general, eukaryotes show a similar pattern, while fungi, as expected, show a much weaker influence of fraction on the community.

10.1128/mBio.02785-19.1 FIG S1 CAP analysis of the prokaryotic, eukaryotic, and fungal communities. The CAP plot was created using “fraction” and “plant species” as factors for Bawburgh and Wytham soil communities. Download FIG S1, PDF file, 0.1 MB. Copyright © 2020 Tkacz et al. 2020 Tkacz et al. This content is distributed under the terms of the Creative Commons Attribution 4.0 International license.

10.1128/mBio.02785-19.10 TABLE S1 Sheet 1, PERMANOVA output for the microbiota structure of four plant species (plant species) grown in two different soils (soil) and sampled using four fractions (bulk soil, rhizosphere, rhizoplane, and root associated). Sheet 2, differences between groups (fraction, soil type, and plant species) of samples analyzed by global ANOSIM of the prokaryotic, eukaryotic, and fungal microbiota. Sheet 3, differences between groups (soil type origin of each fraction) of samples from global ANOSIM of the prokaryotic, eukaryotic, and fungal microbiota. Sheet 4, differences between groups (plant species for each soil type and sampling fraction) of samples from global ANOSIM of the prokaryotic, eukaryotic, and fungal microbiota. Sheet 5, SIMPER output for the experiment to examine the *Medicago* sp. microbiota over a 5-week time course. Sheet 6, ANOSIM values for the *M. truncatula* phyllosphere community structure on transplantation from Bawburgh to Wytham soil and *vice versa*. Sheet 7, Bash and Python scripts giving the annotated code used to analyze next-generation sequencing (NGS) data. Sheet 8, a list of samples for which sequencing reads are archived at the European Nucleotide Archive (ENA) server. Sheet 9, prokaryotic zOTU table presenting zOTU abundances for each biological sample. Sheet 10, eukaryotic zOTU table presenting zOTU abundances for each biological sample. Sheet 12, fungal zOTU table presenting zOTU abundances for each biological sample. Download Table S1, XLSX file, 1.4 MB. Copyright © 2020 Tkacz et al. 2020 Tkacz et al. This content is distributed under the terms of the Creative Commons Attribution 4.0 International license.

**FIG 2:**
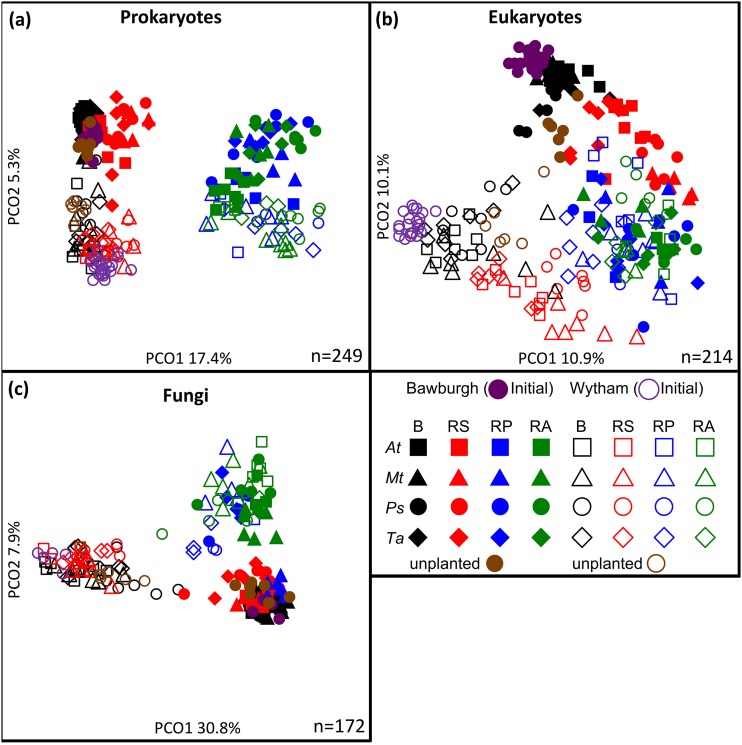
(a to c) Principal-coordinate analysis of the microbiota structure of prokaryotic (a), eukaryotic (b), and fungal (c) communities established by *A. thaliana* (*At*), *M. truncatula* (*Mt*), *P. sativum* (*Ps*), and *T. aestivum* (*Ta*) grown in two different soils (Bawburgh and Wytham). *x* and *y* values show the percent variation explained by each axis. B, bulk soil; RS, rhizosphere; RP, rhizoplane; RA, root associated; *n*, number of samples used in the analysis.

Visualizing the results using principal-coordinate analysis (PCoA) plots (Fig. 2) showed that the first axis (PCO1), which can be attributed to the fraction, explained 17.4% and 10.9% of the total variation for prokaryotes and eukaryotes, respectively (Fig. 2a and b). Initial, unplanted, and bulk soils cluster, while rhizoplane and root-associated fractions cluster. The rhizosphere’s eukaryotic community occupies an intermediate position between soil and root fractions, but its prokaryotic community is closer to bulk soil. The second axis, PCO2, explaining 5.3% and 10.1% of prokaryote and eukaryote data variations, respectively, can be attributed to the soil. While the case for fungi is similar in that the microbiotas from plant-associated fractions form a cluster, the first axis, PCO1 (30.8% variation explained), can be attributed to the soil, i.e., whether the plant is grown in Bawburgh or Wytham soil (Fig. 2c).

In summary, the distance from the root is the predominant influence on the structure of the prokaryotic microbiota of all plants. Soil is very important in the bulk soil and the rhizosphere, while plant species shape microbiotas with different strengths depending on the fraction and soil. Similar microbiotas were established in the plant-associated fractions by diverse plants, regardless of the starting community from the soil in which the plants are grown. As the plants used in this study are taxonomically distant from each other, this suggests conserved mechanisms for the recruitment of plant microbiota.

### Common core of *Proteobacteria* and fungi colonize roots.

**FIG 3:**
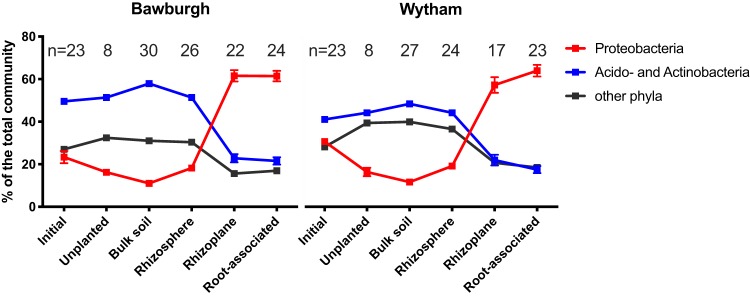
Microbiota taxonomic transition from *Acidobacteria* and *Actinobacteria* to *Proteobacteria* between soil and plant fractions (for a detailed taxonomic profile, please refer to Fig. S2a).

In both Bawburgh and Wytham soils, there are clear differences in the taxonomic structures of the prokaryotic microbiota, with soil and rhizosphere fractions being very different from the rhizoplane and root-associated fractions (Fig. 3 and S2a). *Acidobacteria* and *Actinobacteria* dominate soil and rhizosphere fractions with abundances of 49.6/41.0% (Bawburgh/Wytham) in initial soil, 51.4/44.2% in unplanted soil, 57.9/48.4% in bulk soil, and 51.4/44.2% in the rhizosphere. However, the root fractions are dominated by *Proteobacteria*, which make up 61.6/57.2% (Bawburgh/Wytham) and 61.5/64.0% of the rhizoplane and root-associated fractions, respectively. This occurs for all four plant species grown in two soils, suggesting coselection between plants and *Proteobacteria*. There is some soil-type specificity; Bawburgh-grown plants have more *Alphaproteobacteria* and *Gammaproteobacteria* (47% [standard error {SE}, 3.3%] versus 36% [SE, 3.5%], for Bawburgh- and Wytham-grown plants, respectively; *t* test, *P* < 0.05), while Wytham-grown plants have more *Betaproteobacteria* (13% [SE, 3.3%] versus 29% [SE, 1.1%], for Bawburgh- and Wytham-grown plants, respectively; *t* test, *P* < 0.05) (Fig. S2a). The abundances of other prokaryotic phyla are also different between the soil and root fractions; the soil has higher relative abundances of *Chloroflexi*, *Verrucomicrobia*, and *Thaumarchaeota*. This taxonomic divide can also be observed at the genus level, where Gaiella, Solirubrobacter, and the Spartobacteria class dominate the soil and rhizosphere, while Rhizobium, Sinorhizobium, Sphingomonas, Lysobacter, and Pseudomonas spp. colonize the roots (Fig. S3a). To validate our findings, we have created a global analysis of genus enrichment and depletion patterns across fractions, soils, and plant species. There is more plant species specificity in prokaryotic genus selection in the rhizoplane and root-associated fraction than in the bulk soil and rhizosphere (Fig. S4).

**FIG 4:**
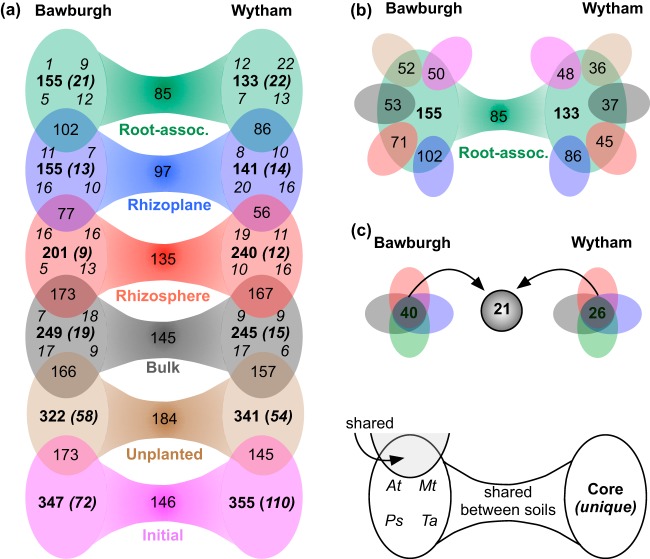
Comparison of the core prokaryotic zOTUs of each plant. (a) Six fractions of four plant species grown in two soils. The core microbiota of each plant consists of common zOTUs found in at least half of the biological replicates. Each dumbbell (fraction) has two ovals for each soil. Each oval represents a number of shared zOTUs between at least 3 plant species (bold), number of zOTUs uniquely found in this fraction (bold italics), and number of zOTUs that are plant species specific (indicated around oval edges; top left, *A. thaliana* [*At*]; top right, *M. truncatula* [*Mt*]; bottom left, *P. sativum* [*Ps*]; and bottom right, *T. aestivum* [*Ta*]). The connecting bridges between the ovals indicate the number of zOTUs shared between soils for each fraction, while the overlapping regions between dumbbell ovals indicate a number of zOTUs shared between fractions. (b) For the root-associated (root-assoc.) fraction, the numbers of shared zOTUs with the initial soil, unplanted soil, bulk soil, rhizosphere, and rhizoplane are indicated within smaller ovals around the main graph using the corresponding color scheme. (c) Number of zOTUs that are found in all the fractions (bulk, rhizosphere, rhizoplane, and root associated) for each soil. The number in the middle represents the number of zOTUs that are shared between two soils. The numbers of plant replicates in Bawburgh and Wytham soils (respectively) are as follows: initial, 18 and 23; unplanted, 8 and 7; bulk soil, 8 and 7 (*At*), 8 and 7 (*Mt*), 8 and 5 (*Ps*), and 6 and 8 (*Ta*); rhizosphere, 7 and 5 (*At*), 5 and 6 (*Mt*), 7 and 5 (*Ps*), and 7 and 8 (*Ta*); rhizoplane; 5 and 3 (*At*), 5 and 6 (*Mt*), 6 and 3 (*Ps*), and 6 and 5 (*Ta*); root associated, 6 and 5 (*At*), 6 and 8 (*Mt*) 6 and 6 (*Ps*), and 6 and 4 (*Ta*). Bar graphs presenting community abundance and taxonomic profile for each of the zOTU groups can be found in Fig. S4.

10.1128/mBio.02785-19.2 FIG S2 Taxonomic structure of the prokaryotic (a), eukaryotic (b), and fungal (c) microbiotas of plants grown in Bawburgh and Wytham soils. A, *A. thaliana*; M, *M. truncatula*; P, *P. sativum*; T, *T. aestivum*; I, initial soil; U, unplanted soil; B, bulk soil; RS, rhizosphere; RP, rhizoplane; RA, root associated. The number of biological replicates is as shown in the legends for Fig. 4 andS3. Download FIG S2, PDF file, 0.1 MB. Copyright © 2020 Tkacz et al. 2020 Tkacz et al. This content is distributed under the terms of the Creative Commons Attribution 4.0 International license.

10.1128/mBio.02785-19.3 FIG S3 (a) Prokaryotic community profile showing the most abundant genera. (b) Eukaryotic community profile showing taxa best separating the microbial fractions (based on the SIMPER analysis). Each fraction is split using four bars; from the left, *A. thaliana, M. truncatula, P. sativum*, and *T. aestivum*. (c and d) Comparison of the core eukaryotic (c) and fungal (d) OTUs of each plant. This is shown for all fractions, soils, and plants. The core microbiota of each plant consists of common zOTUs found in at least 40% of the biological replicates. Each dumbbell (fraction niche) has two ovals for each soil. Each oval represents a number of shared zOTUs between at least 3 plant species (bold), number of zOTUs uniquely found in this niche (bold italics), and number of zOTUs that were plant species specific (indicated around oval edges; top left, *A. thaliana* [*At*]; top right, *M. truncatula* [*Mt*]; bottom left, *P. sativum* [*Ps*]; and bottom right, *T. aestivum* [*Ta*]). The connecting bridges between the ovals indicate the numbers of zOTUs shared between soils for each niche, while the overlapping regions between dumbbell ovals indicate number of zOTUs shared between niches. (e and f) For eukaryotes (e) and fungi (f), for the root-associated fraction, the numbers of shared zOTUs with the initial soil, unplanted soil, bulk soil, rhizosphere, and rhizoplane are indicated within smaller circles around the graph using the corresponding color scheme. (g and h) For eukaryotes (g) and fungi (h), the numbers of zOTUs that are found in all of the fractions (bulk, rhizosphere, rhizoplane, and root associated) for each soil. The numbers in the middle represent the number of zOTUs that are shared between two soils. The bar graphs presenting community abundances and taxonomic profiles for each of the zOTU groups can be found in Fig. S5 and S6. I, initial soil; U, unplanted control; B, bulk soil; RS, rhizosphere; RP, rhizoplane; RA, root associated. The numbers of plant replicates per bulk soil, rhizosphere, rhizoplane, and root-associated fractions (in Bawburgh and Wytham soils, respectively) are as follows: for eukaryotes, bulk 8 and 7 (*At*), 8 and 6 (*Mt*), 8 and 6 (*Ps*), and 6 and 8 (*Ta*); for rhizosphere, 7 and 5 (*At*), 5 and 5 (*Mt*), 8 and 5 (*Ps*), and 7 and 8 (*Ta*); for rhizoplane, 1 and 2 (*At*), 1 and 6 (*Mt*), 5 and 1 (*Ps*), and 6 and 5 (*Ta*) (27 total); for root-associated, 2 and 2 (*At*), 4 and 3 (*Mt*), 4 and 5 (*Ps*), and 4 and 2 (*Ta*) (26 total); 7 and 6 (unplanted), 22 and 15 (initial); for fungi, bulk 6 and 8 (*At*), 8 and 7 (*Mt*), 8 and 4 (*Ps*), and 8 and 7 (*Ta*); for rhizosphere, 6 and 3 (*At*), 3 and 5 (*Mt*), 8 and 4 (*Ps*), and 6 and 7 (*Ta*); for rhizoplane, 0 and 0 (*At*), 5 and 4 (*Mt*), 3 and 2 (*Ps*), and 3 and 3 (*Ta*); for root-associated, 2 and 3 (*At*), 6 and 6 (*Mt*), 5 and 6 (*Ps*), 1 and 1 (*Ta*); 8 and 7 (unplanted), and 4 and 5 (initial). Download FIG S3, PDF file, 0.2 MB. Copyright © 2020 Tkacz et al. 2020 Tkacz et al. This content is distributed under the terms of the Creative Commons Attribution 4.0 International license.

10.1128/mBio.02785-19.4 FIG S4 Prokaryotic genus-level selection and depletion analysis. For each fraction and soil, prokaryotic genera are indicated to be either selected or depleted (above or below the *x* axis, respectively) by an individual plant species. Genera (434 in total) are plotted alphabetically; however, only a selected few are indicated. Download FIG S4, PDF file, 1.1 MB. Copyright © 2020 Tkacz et al. 2020 Tkacz et al. This content is distributed under the terms of the Creative Commons Attribution 4.0 International license.

The taxonomy of the eukaryotic community is dominated by the fungal phylum of Ascomycota and, to a lesser extent, Basidiomycota. Ascomycota dominate all fractions, averaging 42.1%/41.4% (SE, 1.0% and 0.3%, respectively) (Bawburgh/Wytham), with Basidiomycota accounting for 7.3%/6.1% (SE, 1.2% and 0.2%, respectively) of the total eukaryotic populations (Fig. S2b). Rhizosphere and root fractions in plants grown in both soils have a high percentage of Metazoa, which notably comprises approximately 30% (SE, 0.03%) of the *M. truncatula* rhizosphere. The protist group (Stramenopiles, Alveolata, and Rhizaria) and Amoebozoa, as well as green algae, Chlorophyta, were found across all fractions. Based on the SIMPER analysis, the differences in the abundances of the Metazoa, especially of Nematoda and Panarthropoda, are dominant in separating the eukaryotic fractions. Other taxa responsible for their separation are the Alveolata phyla of Apicomplexa and Ciliophora, which have high abundances in rhizosphere and rhizoplane fractions. In addition, the fungal groups of Fusarium and Ustilaginomycotina are abundant in the plant-associated fractions, as well as Oomycetes found predominantly in the rhizosphere (Fig. S3b).

The fungal community was analyzed at the genus level (Fig. S2c), and apart from Lactarius, all dominant genera belong to the phylum Ascomycota. The greatest difference in the communities is the increasing abundance of the genus *Stachybotrys* in the roots of all plants, in both soils. While this genus is found at less than 1% relative abundance (SE, 0.04%) in unplanted and bulk soil, it rises to approximately 20% (SE, 1.4%) in the rhizoplane and root-associated communities. *Stachybotrys* spp. are known cellulose degraders mainly found in damp wooden walls (10) and wetlands (11). This genus aside, the fungal community of Bawburgh samples is conserved throughout the different fractions and plant species. However, Wytham samples show suppression of Paraconiothyrium, Chaetomium, and Myrmecridium spp. These organisms are found in initial soil but are lost in the unplanted control and under plant growth; hence, we speculate that these genera were outcompeted in soil under glasshouse conditions (Fig. S2c).

In summary, the plant-associated microbiota is similar at the phylum level but becomes more distinct at lower taxonomic levels. *Proteobacteria* are dominant in plant roots, while the soil influences the classes of *Proteobacteria* present. Nematoda and Metazoa dominate the rhizosphere and are found in root fractions. Fungi are the dominant eukaryotic group, with cellulose-degrading *Stachybotrys* spp. outcompeting other fungi in the colonization of plants under our growth conditions.

### All plant species are colonized by the same soil microbiota, but they are able to shape its structure.

In order to understand the dynamics of microbiota colonization of plants, initial, unplanted, and bulk soil, rhizosphere, rhizoplane, and root-associated microbiota prokaryotic communities were divided by soil (Fig. 4a). Microbial zero-radius operational taxonomic units (zOTUs) were found in at least half of the biological replicates, and at least three out of the four plant species were considered core zOTU members for a given fraction. Core zOTUs for each fraction were compared with those of neighboring fractions as well as against their counterparts grown in the other soil. Analysis of the prokaryotic community demonstrates the convergence of the rhizoplane and root-associated fractions. In both soils, the number of core zOTUs decreases from approximately 350 in initial and unplanted soils to approximately 250 in bulk soil and the rhizosphere before further decreasing to approximately 150 zOTU in the rhizoplane and root fractions. The number of core zOTUs that are unique to each fraction are 72 to 110, depending on the soil (from a total of approximately 350) for initial soil but fall to approximately 10 to 20 (from a total of approximately 150 to 250) for fractions closer to the root, reflecting the fall in the fraction of the microbiota that are unique. Bar graphs showing the taxonomic profile of the core zOTUs reveal changes at the phylum level of the microbiota (Fig. S5). These clearly illustrate plant colonization by specific zOTUs. The taxonomies of core rhizoplane and root-associated zOTUs are strikingly similar to each other, with a high proportion of *Proteobacteria* (however, the two soils differ in proportions of proteobacterial classes). Core microbiotas of soil fractions and rhizosphere are dominated by *Acidobacteria*, *Actinobacteria*, *Chloroflexi*, and *Thaumarchaeota* (archaea) (Fig. S5a).

10.1128/mBio.02785-19.5 FIG S5 Taxonomic profiles of prokaryotic zOTUs groups. For each of the zOTU groups identified in Fig. 4, a taxonomic profile is presented with the percentage of relative abundance. (a) As an example, bar plot “I,” on the left, represents 34% of the community, which indicates that 34% of the total zOTU abundance was shared between replicates of the initial soil samples (while 66% of the community was found in some samples but was not shared by more than 50% of the replicates). zOTU group profiles for fraction cores and shared between fractions and soils, and below are zOTUs found only in specific fraction or plant species (unique) (as in Fig. 4a). (b) zOTU group profiles of community shared between each fraction and the root-associated fraction (as in Fig. 4b). (c) Core microbiotas shared between all fractions (bulk, rhizosphere, rhizoplane, and root associated) corresponding to Fig. 4c. I, initial soil; U, unplanted control; B, bulk soil; RS, rhizosphere; RP, rhizoplane; RA, root associated. Download FIG S5, PDF file, 0.1 MB. Copyright © 2020 Tkacz et al. 2020 Tkacz et al. This content is distributed under the terms of the Creative Commons Attribution 4.0 International license.

While these two groups of taxonomic profiles (i.e., soil/rhizosphere versus rhizoplane/root associated) appear to bear little resemblance to each other, it is clear from the quantitative overlap between the core zOTUs of the fractions (Fig. 4b) that many of the zOTUs found inside the root (root-associated microbiota) are derived from that of the soil in which the plant is grown. There is a substantial and, in general, an increasing percentage overlap of the root-associated prokaryotic core zOTUs with those of initial, unplanted, and bulk soil, rhizosphere, and rhizoplane (Fig. 4b). For the soil fractions, these overlaps were dominated by *Acidobacteria*, *Actinobacteria*, *Chloroflexi*, and *Thaumarchaeota*, which, while common in soil, can still be found inside the roots. The rhizosphere, and especially the rhizoplane, have a substantial proportion of their proteobacterial communities shared with the plant root (Fig. S5b).

There were a number of prokaryotic zOTUs that are plant species specific for a given soil and fraction (Fig. 4a). However, their number is small relative to the common fraction zOTU pool and their relative abundance low (Fig. S5a). Individual plant species do have significant differences in their microbiotas, but PCoA and PERMANOVA show that it is primarily due to altered abundance rather than the presence or absence of particular microbial species.

A small number of zOTUs can be found to be stably associated with all fractions (bulk soil, rhizosphere, rhizoplane, and root associated). For Bawburgh soil, there are 40 zOTU, and for Wytham soil, there are 26 zOTU fulfilling these criteria, of which 21 are shared between these groups (Fig. 4c). These core communities are not rich in diversity; however, they represent a relatively abundant part of the overall community (Fig. S5c).

Eukaryotic and fungal microbiotas were compared in the same way (Fig. S3c and d). The overall conclusions that can be drawn for eukaryotes and fungi are as for the prokaryotic community; plants are colonized by a common core community from the soil, despite differences in the microbiotas of initial soil (especially in fungi). The majority of zOTUs found inside roots are found across different fractions (Fig. S3e and f). In addition, the richness decreases the closer the fraction is to the root, although the clear-cut taxonomic division seen in the prokaryotic communities, between rhizoplane and root-associated core zOTUs and those of the soil and rhizosphere, was not observed (Fig. S6 and S7). Individual plant species, as in case of the prokaryotic community, did not attract unique zOTUs in any of the analyzed fraction (Fig. S3c and d, S6a, and S7a). In the case of the eukaryotic community, about half of the microbiota members can be found in all fractions (48% [84 zOTU] and 51% [99 zOTU] for Bawburgh and Wytham soil, respectively) (Fig. S3g). The fungal core microbiotas are even more abundant across all fractions, with the Bawburgh core representing 86% (34 zOTUs) of the total community and the Wytham core representing 70% (29 zOTU) of the total community (Fig. S3h).

10.1128/mBio.02785-19.6 FIG S6 Taxonomic profiles of eukaryotic zOTU groups. For each of the zOTU groups identified in Fig. S3c, a taxonomic profile is presented with the percentage of relative abundance. (a) As an example, bar plot “I,” on the left, represents 63% of the community, which indicates that 63% of the total zOTU abundance was shared between replicates of the initial soil samples (while 37% of the community was found in some samples but was not shared by more than 50% of the replicates). zOTU group profiles for fraction cores and shared between fractions and soils, and below are zOTUs found only in specific fraction or plant species (unique) (as in Fig. S3c). (b) zOTU group profiles of communities shared between each fraction and the root-associated fraction (as in Fig. S3e). (c) Core microbiotas shared between all fractions (bulk, rhizosphere, rhizoplane, and root associated) corresponding to Fig. S3g. I, initial soil; U, unplanted control; B, bulk soil; RS, rhizosphere; RP, rhizoplane; RA, root associated. Download FIG S6, PDF file, 0.1 MB. Copyright © 2020 Tkacz et al. 2020 Tkacz et al. This content is distributed under the terms of the Creative Commons Attribution 4.0 International license.

10.1128/mBio.02785-19.7 FIG S7 Taxonomic profiles of fungal zOTU groups. For each of the zOTU groups identified in Fig. S3d, a taxonomic profile is presented with the percentage of relative abundance. (a) As an example, bar plot “I,” on the left, represents 92% of the community, which indicates that 92% of the total zOTU abundance was shared between replicates of the initial soil samples (while 8% of the community was found in some samples but was not shared by more than 50% of the replicates). zOTU group profiles for fraction cores and shared between fractions and soils, and below are zOTUs found only in specific fraction or plant species (unique) (as in Fig. S3d). (b) zOTU group profiles of communities shared between each fraction and the root-associated fraction (as in Fig. S3f). (c) Core microbiotas shared between all fractions (bulk, rhizosphere, rhizoplane, and root associated) corresponding to Fig. S3h. I, initial soil; U, unplanted control; B, bulk soil; RS, rhizosphere; RP, rhizoplane; RA, root associated. Download FIG S7, PDF file, 0.1 MB. Copyright © 2020 Tkacz et al. 2020 Tkacz et al. This content is distributed under the terms of the Creative Commons Attribution 4.0 International license.

### Plant influence reduces microbial diversity.

We used the Shannon diversity index to determine microbiota diversity. In general, bulk soil and rhizosphere increase, while rhizoplane and root-associated fractions decrease the prokaryotic and fungal diversity compared with unplanted soil controls (*t* test with Bonferroni correction, *P* < 0.05). However, the eukaryotic community diversity does not change in different fractions. For the fungal community, the diversity is more dependent on soil than for either prokaryotic or general eukaryotic communities (Fig. S8). It is possible that the measured diversity may be affected by the total number of microbial cells sampled for each fraction; however, we show that all fractions contain a rich microbiota. It is only by extremely deep sequencing that it may possible to observe a connection between sample microbiota load and its diversity.

10.1128/mBio.02785-19.8 FIG S8 Shannon diversity index calculated for prokaryotic, eukaryotic, and fungal communities of each fraction for Bawburgh and Wytham soils. * indicates a significant difference against unplanted control calculated using a *t* test with Bonferroni correction for multiple testing. *n*, number of replicates as detailed in Fig. 1. Download FIG S8, PDF file, 0.1 MB. Copyright © 2020 Tkacz et al. 2020 Tkacz et al. This content is distributed under the terms of the Creative Commons Attribution 4.0 International license.

### Time course of microbial community development.

**FIG 5:**
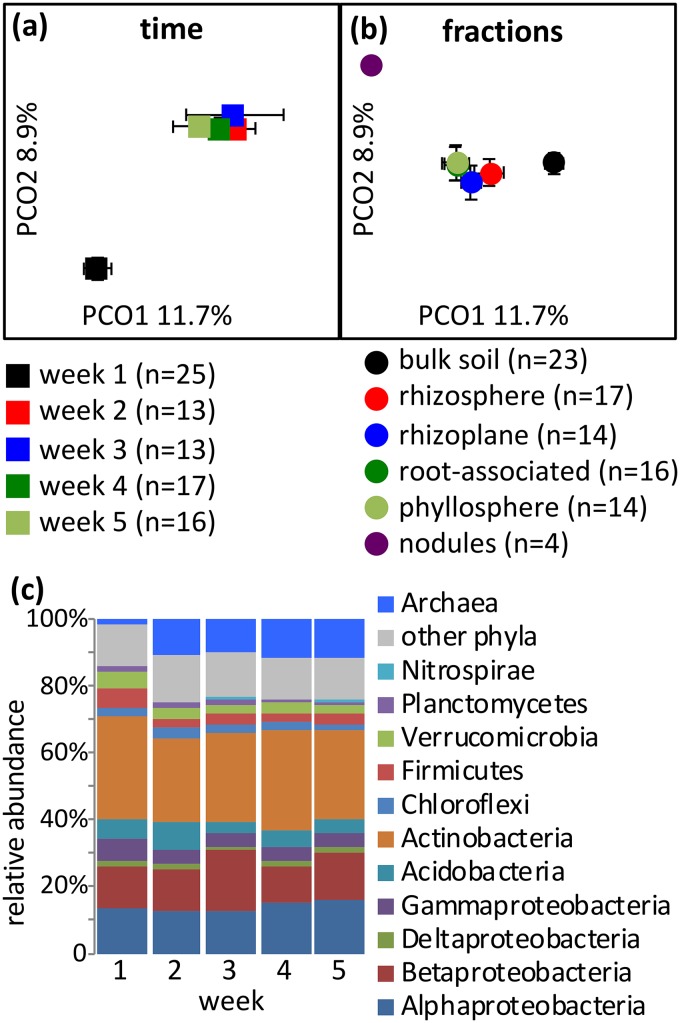
The microbiota structure changes over time and is dependent on the plant fraction. (a) Principal-coordinate analysis of prokaryotic microbiota structure considered by time (samples taken over 5 weeks [weeks 1 to 5]) from *M. truncatula* grown in Bawburgh soil (all fractions; bulk soil, rhizosphere, rhizoplane, root associated, and phyllosphere, but excluding nodules). (b) Prokaryotic microbiota considered by plant fraction (samples taken over 5 weeks [weeks 1 to 5]) from *M. truncatula* grown in Bawburgh soil. Nodule samples were only taken at week 5. (c) Taxonomic structure of the total plant microbiota (excluding nodules) of *M. truncatula* grown in Bawburgh soil over 5 weeks, with the week of sampling indicated. (and b) Error bars indicate the variation between individual samples (for a detailed representation, see Fig. S9), n, number of biological replicates.

Since all community structures were determined in 4-week-old plants, it is important to know whether the communities were stable or still in transition at this time. To address this, the colonization of *M. truncatula* was analyzed weekly for 5 weeks. A single plant species was used for practical reasons; however, as it had already been established that 4-week-old plants of different species have broadly similar microbiotas, we tested whether this observation might depend on sampling age. For *Medicago* spp., the analysis was expanded to include the microbiotas of both the phyllosphere and nitrogen-fixing nodules formed on the roots of this legume. The root microbiotas were distinct after 1 week, had stabilized by the second week, and became relatively stable over subsequent weeks (Fig. 5a and S9a and b). This was confirmed by PERMANOVA, with samples from all time points having a strong, significant difference (pseudo-F, 3.26; *P* < 0.001); while PERMANOVA conducted after *in silico* removal of samples from the first week was still significant, it indicated a reduced community separation (pseudo-F, 1.34; *P* = 0.008). Based on the SIMPER analysis, archaea rather than any bacterial phyla were predominantly responsible for the weekly shift in the microbiota (Fig. 5c and Table S1, sheet 5).

10.1128/mBio.02785-19.9 FIG S9 (a) Microbiota dynamics shown using PCoA plots using all the data. (b and c) Biological replicates binned into single data points, where error bars show the standard error of the PCoA location (b), and data binned according to the fraction (c). (a to c) The number of biological replicates is indicated next to the legend symbols. (d) Phylogenetic profile at the genus level of microbiotas changing over time and plant fractions. Only the abundances of the top 16 genera are presented for visual clarity (out of 434 genera observed throughout the experiment). (e) Relative abundance of the *Rhizobiaceae* family across fractions, with the data shown using a box plot with 9th to 95th percentile whiskers. I, initial soil; U, unplanted control; B, bulk soil; RS, rhizosphere; RP, rhizoplane; RA, root associated. The number of replicates (*n*) is indicated next to the symbol description. Download FIG S9, PDF file, 0.3 MB. Copyright © 2020 Tkacz et al. 2020 Tkacz et al. This content is distributed under the terms of the Creative Commons Attribution 4.0 International license.

As we used *M. truncatula* in this experiment, we looked into the abundances of *Rhizobiales* genera, which includes *Rhizobium*, *Sinorhizobium*, and Bradyrhizobium. These genera increased in abundance over time in all sampled fractions (Fig. S9d).

To examine the nodule microbiota, nodules were rinsed to remove any adhering soil but not surface sterilized. As expected, nodules of *M. truncatula* are dominated by its cognate microsymbiont *Sinorhizobium*, but it also had a significant abundance of Azohydromonas spp. (Fig. S9d). As it is difficult to sonicate or vortex nodules without releasing rhizobial symbionts from inside nodules, we did not try to separate the communities on the nodule surface from those inside them. As *Sinorhizobium* spp. form nodules on this legume host, we can be confident that these are primarily within the plant cells of the nodule, while it is possible that *Azohydromonas* spp. may be solely on the nodule surface. The dominant annotation obtained for the *Azohydromonas* zOTU is A. australica DSM 1124 (12), a strain reclassified from hydrogen-oxidizing Alcaligenes latus (13), that grows using CO_2_, H_2_, and N_2_ as sole carbon, energy, and nitrogen sources. Nitrogenase activity within nitrogen-fixing nodules formed by *Sinorhizobium* spp. generates freely diffusing H_2_ (albeit, the precise concentration depends on the presence or absence of rhizobial bacteroid uptake hydrogenases), giving *Azohydromonas* spp. an advantage over other nodule surface colonizers. While a significant amount of members of this genus was found in the rhizosphere and rhizoplane, which may also be enriched with H_2_, other fractions had only minor numbers of *Azohydromonas* spp. (Fig. S9d).

### Origin of the phyllosphere microbiota.

**FIG 6:**
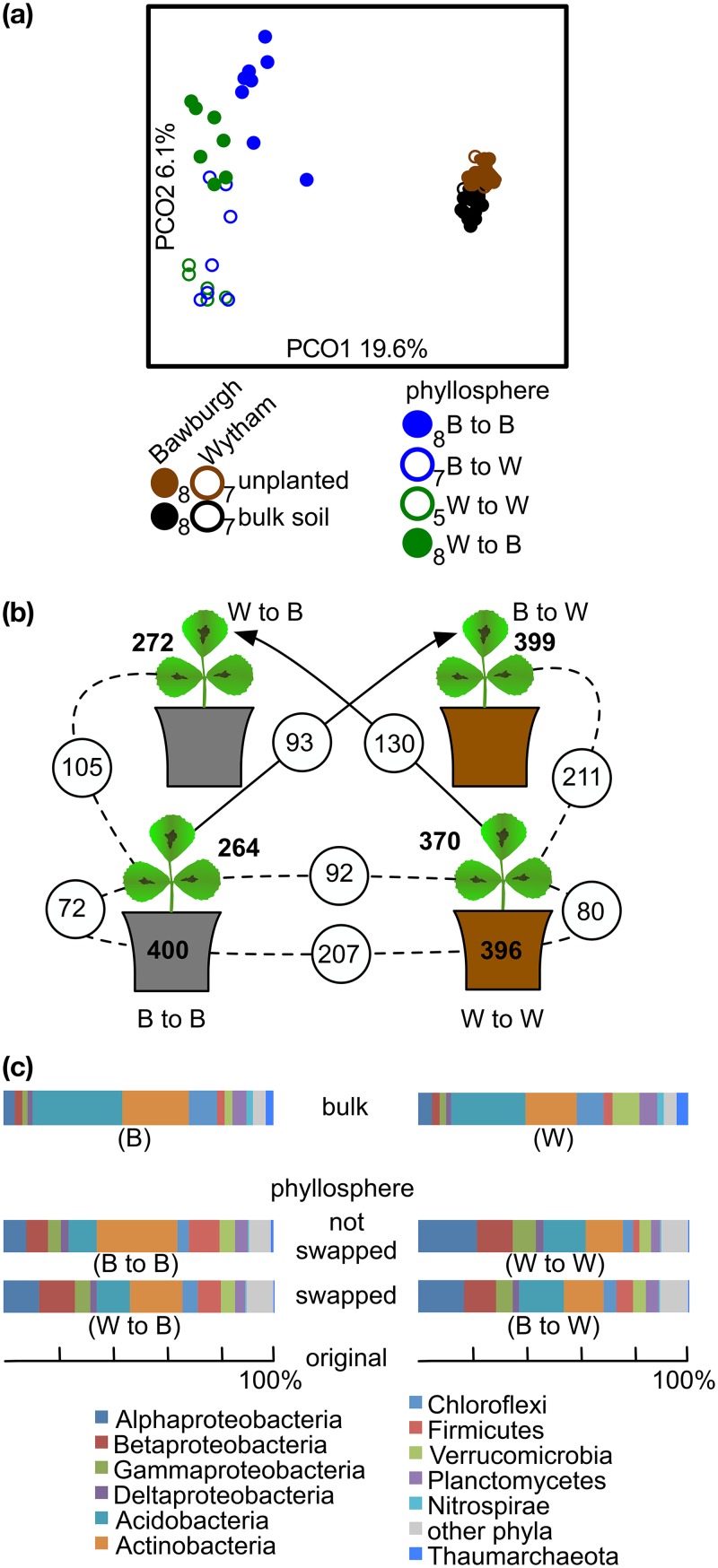
Similarity of the microbiota structures of the phyllosphere of transplanted *M. truncatula* plants with those of the soil in which they are growing. Plants were grown in Bawburgh (B) soil and transplanted into Bawburgh (B to B) and Wytham (B to W) soils, or grown in Wytham (W) soil and transplanted into Bawburgh (W to B) and Wytham (W to W) soils. For comparison, the microbiotas of unplanted and bulk soil community structures are shown. (a) PCoA plot representing the total microbiota structure. (b) Diagram representing the number of core zOTUs found in bulk soil (on pot) in which *M. truncatula* plants were grown and phyllosphere (core zOTUs must be found in at least half of biological replicates); numbers in circles show zOTUs shared between soils, soil and phyllosphere, and between phyllospheres. (c) Bar graphs represent phylum community structures of the bulk soil (B or W), phyllosphere of plants which were not swapped (B to B and W to W) and swapped (W to B and B to W). (a) The number of biological replicates is provided next to the legend symbols.

Analysis over 5 weeks showed that the microbiota structure of the phyllosphere is similar to those of the rhizoplane and root-associated fractions (Fig. 5b and S9c). It has already been reported for poplar that the phyllosphere microbiota profile is similar to the root-associated microbiota profile (14). Hence, we speculated that the phyllosphere microbiota consists mainly of the soilborne taxa best able to colonize plants. In order to understand this colonization further, a soil swap experiment was performed. *M. truncatula* plants were initially grown for 2 weeks either in Bawburgh or Wytham soil, as this allows the root and phyllosphere microbiota to stabilize. After 2 weeks, plants were transplanted either into the reciprocal soil or back into the original one. Plants were grown for a further 2 weeks. Community structure visualization using PCoA and statistical analysis using ANOSIM indicate that the phyllosphere microbiota of the majority of plants moved from Bawburgh to Wytham soil closely resembles that of plants grown in Wytham soil for 4 weeks, while that of plants transplanted from Wytham into Bawburgh soil had an intermediate structure (Fig. 6a and Table S1, sheet 6). Changes in the phyllosphere microbiota structure after plants were moved to new soil indicate that plant leaves were recolonized by the microbes present in the recipient soil. Bulk soils of Bawburgh and Wytham harbor a similar number of prokaryotic zOTUs (∼400 each). However, in their phyllosphere fraction, plants grown in Bawburgh soil support only 264 zOTU, while those grown in Wytham soil have a richer microbiota structure of 370 zOTU (Fig. 6b). Remarkably, the phyllosphere of Bawburgh soil-grown plants when moved to Wytham soil became enriched to harbor 399 zOTU, of which 211 are found in Wytham soil, while the opposite happened to those plants from Wytham soil moved into Bawburgh soil, reducing their richness to 272 zOTU, where 105 of them are found in Bawburgh soil. The recolonization process is not complete, as 93 zOTU are still found on plants moved into Wytham soil with the Bawburgh-grown ones, while for the plants moved from the opposite soil, this number is 130 zOTU. Moreover, the taxonomic structure was also affected by the soil swap, where the phyllosphere of plants moved from one soil to the other (either Bawburgh to Wytham or *vice versa*) became more similar to the phyllosphere of plants from the second soil (Fig. 6c).

## DISCUSSION

Plant microbiotas have recently been studied intensively to determine the influences of the plant host species and genotype (8, 9, 15). Confusingly, the microbiota is also affected by different growth locations, soils, and environmental stresses (16). Studies on single plant species have shown that the rhizosphere is different from bulk soil and that the root has a very distinct microbiota (17). There is support for a hypothesis that plants coevolved with their prokaryotic microbiota, as shown by screening the roots of many wild plants grown in nature (18). This study showed the interplay of soil type and plant species factors shaping the microbiota structure; however, due to the fact that plants were sampled in a natural environment (with sampling site-to-site geochemical composition, moisture, insect, etc. differences), it is difficult to separate these factors with statistical precision. A study by Yeoh et al. showed only a small percentage increase in *Proteobacteria* abundance in the roots compared to the soil (18), while our controlled sampling procedure allowed for a detailed bulk soil/rhizosphere versus rhizoplane/root microbiota community separation, and we show that different plant families establish remarkably similar, *Proteobacteria*-rich root microbiotas, even from contrasting soils (Fig. 2 and 3). Our work may help explain contradicting findings in the literature, where sometimes, different plant species establish different microbial root communities (see, for example, reference 19), and sometimes these communities are very similar, especially for closely related plant species (20). As the fraction factor exerts approximately 4 times stronger influence on the prokaryotic microbiota structure than the plant species (much smaller differences in the case of eukaryotes and an inverted relationship for fungi) (Fig. 1), it becomes obvious that difference in sampling procedures may be critical. Potentially, plants with a very similar root microbiota but different root architecture may produce a false dissimilarity in their community structures due to the fact that different amounts of soil or microbial biofilm stick to the roots. The same may be true for soils where different amounts of tiny clay minerals may strongly adhere to the roots, affecting the results.

All of the plants selected for the same prokaryotic, eukaryotic, and fungal zOTUs; however, their relative abundances were influenced by the plant species (Fig. 4 and S3c and d). This similarity can best be observed by growing multiple plant species at the same time in different soils and comparing the microbiotas of root-associated, rhizoplane, rhizosphere, and bulk soil fractions. Moreover, we analyzed the total plant microbiotas of bacteria, archaea, microbial eukaryotes, soil-dwelling animals, nematodes, and fungi. Due to this broad approach, we can conclude that there are both conserved mechanisms of selection in plants and mechanisms of adaptation in microbes. There are likely to be evolutionarily conserved plant and bacterial genes that are responsible for microbiota recruitment and cohabitation, as shown using genome screening of thousands of bacterial strains (21). Root exudation and plant immunity are likely to be crucial in allowing genetically similar microorganisms to colonize the rhizoplane and root (22). It was shown that immunity-impaired *Arabidopsis* plants are colonized by a different bacterial community from that of wild-type plants (23). However, although, as expected, there are significant plant species-specific effects, their individual effect is much smaller than that of the fraction, followed by the effect of the soil (Fig. 1 and Table S1, sheet 1). This of course does not preclude some plant species from having much stronger effects on their microbiota, particularly given the diversity of plant immunity, secondary metabolism, and root secretion. Rather, we show that coselection must be acting for a taxonomically diverse group of plants.

*Proteobacteria* dominate the rhizoplane and root-associated microbiota, while they are less common in the bulk soil and the rhizosphere (Fig. 3). The high proportion of this phylum inside roots was partly associated with lignocellulose abundance (17). However, the finding that this phylum is able to colonize various plants grown under different soil conditions indicates that they are also able to avoid detection by the plant immune response or are able to overcome it. The rhizoplane and the root endosphere are rich in plant-derived nutrients, and *Proteobacteria* with their fast metabolism may be able to use these resources to outcompete other bacteria (24). Conversely, root-associated and rhizoplane microbiotas have reduced abundances of *Acidobacteria* and *Actinobacteria*, while these phyla are dominant in the bulk soil and rhizosphere.

Legume and nonlegume plants are colonized by bacteria from the family *Rhizobiaceae*. It was already shown that *Arabidopsis* spp., barley, and Lotus spp. are hosts for these bacteria, as they form a symbiosis with legumes, promote the growth of *Arabidopsis* spp., and potentially support microbial homeostasis preventing pathogen attacks (25). We confirm that *Rhizobiaceae* are a part of the core microbiotas of plants belonging to different families, regardless of the soil in which the plants were grown. Legume plants have substantially more *Rhizobiaceae* than do nonlegume plants, despite the effort to remove all visible nodules during sampling (Fig. S9e).

The rhizosphere and rhizoplane are nutrient-rich niches for nematodes, arthropods, Alveolata, and Oomycetes, while inside the roots, their abundances are reduced. Members of the fungal genus *Stachybotrys* managed to colonize all of the plants examined from both soils. This genus was barely detected in bulk soil or rhizosphere, indicating its ability to evade plant immunity and compete against other microorganisms in penetrating root tissue (Fig. S2c). However, as *Stachybotrys* spp. grow well under damp conditions, their dominance may be highly dependent on the humid growth conditions in a glasshouse as used in this study for plant growth.

Root-colonizing bacteria and eukaryotes are recruited from the surrounding soil. Many bacterial, eukaryotic, and fungal species common in roots can also be found in the plant rhizoplane, rhizosphere, and bulk soil (Fig. 4 and S3c to h), where their selection affects the microbial diversity in each fraction. Prokaryotic and eukaryotic microbiota diversity generally was reduced in the rhizoplane and root-associated fraction. Soil did not affect the prokaryotic or eukaryotic diversity but had a strong influence on fungi (Fig. S8).

In the controlled environment of the glasshouse, plants gain a relatively stable microbiota structure after 2 weeks of growth. Rhizosphere, rhizoplane, and root-associated fractions of *M. truncatula* need the same amount of time to stabilize (Fig. 5 and S9a and b). Hence, data obtained even from a single time point represent a general plant microbiota. In a soil disturbance study, where a soil community recolonized a sterilized soil substrate, the relative proportions of archaea to bacteria rose successively over a 24-week period (26). We have also observed increased archaeal abundance, especially between the first and later weeks in the bulk soil. A study of the *Medicago* microbiota sampled at different vegetative stages over 117 days showed that the major change occurs during the flowering stage (day 70) (27). As we have not sampled at such a late growth stage, we cannot exclude the possibility that the microbiota of the sampled plants would also change at later time points.

The abundance of the hydrogen-oxidizing genus *Azohydromonas* increased over time in the rhizosphere and rhizoplane. *Azohydromonas* was also the second most abundant genus after the N_2_-fixing *Sinorhizobium* genus in nodule samples. We speculate that nitrogen fixation, which releases H_2_, selects for hydrogen-oxidizing bacteria (Fig. S9d).

The phyllosphere microbiota is remarkably similar to the root-associated one and follows the same colonization process. This suggests that the phyllosphere microbiota is similar in structure to the other plant fractions, as it is continuously colonized by the soil microbiota. If the source of this microbiota is changed by swapping soils, the phyllosphere community restructures and resembles the community of the final soil into which it was placed (Fig. 6). Under field conditions, the phyllosphere was also found to closely resemble the soil community (9). Moreover, plastic replicates of plants were colonized by a community very similar to that colonizing surrounding plants (28). It is possible that in the wild, colonization of the phyllosphere is accelerated by wind, rain splashes, and insects; however, the soil itself is the main source for phyllosphere communities. Synthetic bacterial communities derived from soil and leaves were able to colonize the germfree leaves and roots of *Arabidopsis* spp. (29). These communities consisted of around 200 isolated strains that were able to assemble into a very similar structure compared with its native community from which they were isolated. Moreover, there was a significant functional overlap between leaf- and root-derived bacterial community members. Our finding that soil bacteria constantly colonize plant leaves may help explain the reason why communities screened by Bai et al. (29) show structural and functional similarities, as the leaf community could be viewed as part of the wider plant soil and root communities. Moreover, our conclusions regarding colonization patterns are strengthened with the observation of many soilborne *Enterobacteriaceae* being found on the leaves of rucola plants (30).

The main conclusion of this work is that different plants establish very similar plant-associated microbiotas in their rhizoplane and root endosphere. Individual plant species shape relative abundance, rather than presence or absence of the microbiota members. Thus, plants and microbes must use common mechanisms to interact with each other, and this must have been under strong coselection. However, fungal microbiota is predominately influenced by soil rather than the plant. The environmental niches created by plants are fully colonized in 2 weeks, including the phyllosphere that is being constantly colonized by a subset of soilborne bacteria.

## MATERIALS AND METHODS

### Plant growth.

Three independent plant growth experiments were performed using soils from Bawburgh farm and Wytham woods that have been characterized previously (31).

For experiment 1, the microbiota from four plant species covering three agriculturally important families, Brassicaceae (*A. thaliana* Col-0), Leguminosae (*M. truncatula* A17 and the crop legume *P. sativum* var. Avola), and Poaceae (*T. aestivum* var. Paragon), were grown using two different soils, poor Bawburgh and rich Wytham soils of similar pHs under glasshouse conditions, and watered as needed and without further fertilization. Plants were grown using 8 biological replicates. Seeds were surfaced sterilized by using ethanol (70%) and bleach (4%) and germinated on MS agar (32). Plates with microbial contamination were discarded. Seedlings were transplanted into 50-ml pots and grown for 4 weeks. As we compare plant species of different growth timings, we sampled young plants before flowering. Under our glasshouse conditions, *Arabidopsis* spp. start the bolting stage at about 4 weeks. Plants were watered carefully to avoid soil splashes onto the leaves.

For experiment 2, how the microbiota changes over time was determined on *M. truncatula* grown in Bawburgh soil as described above and sampled every week for 5 weeks.

Experiment 3 was performed to determine microbiota recolonization using *M. truncatula* and both soils. Plants were treated as described above until after 2 weeks of growth, when all the plants were removed. Half of the plants grown in Bawburgh soil were transplanted back into Bawburgh soil, and half of them were put into Wytham soil. The same experiment was performed on Wytham soil-grown plants. Plants were moved using gloves, roots were washed, and the shoots were prevented from touching the soil. After this, plants were grown for another 2 weeks before sampling.

### Sampling of soil and plant fractions.

Plants, including their roots, were removed from the pots. Usually, the whole pot would be penetrated with plant roots, and the bulk soil fraction was shaken off to leave a thin coating (1 to 2 mm) of soil adhering to the roots. This thin soil fraction was vortexed off the roots (2,000 rpm for 5 min using a Multi Reax shaker; Heidolph, Schwabach, Germany) to yield the rhizosphere fraction. Next, the root was placed in a 15-ml conical tube and placed in an ultrasonic bath for 5 min at 37 kHz of ultrasonic frequency (model FB15056; Fisherbrand, Loughborough, UK). The released microbiotas were spun down for 10 min at 4,000 rpm to yield the rhizoplane fraction. The root-associated fraction was prepared from sonicated roots crushed in liquid nitrogen. The same procedure was employed for the leaves to sample for the phyllosphere. For legumes, after isolation of the rhizosphere fraction, visible nodules were removed, washed (but not surface-sterilized), and crushed to yield the nodule fraction. Each fraction corresponds to a plant fraction of the same name. For each experiment, we sampled and analyzed biological replicates, and no technical replicates were used. We did not pool any soil, root, or their DNA or PCR samples, but rather, every presented data point (sample) represents a single plant or its surrounding soil.

### DNA isolation, PCR amplification, and sequencing.

DNA was isolated from 300 mg of soil or roots using a ZR Soil Microbe DNA kit (D6001; Zymo Research, Irvine, CA, USA). Protein nucleic acid PCR clamps (1 μM) targeting plastid (pPNA, 5′-GGCTCAACCCTGGACAG-3′) and mitochondrial (mPNA, 5′-GGCAAGTGTTCTTCGGA-3′) DNA (PNA Bio, Newbury Park, CA, USA) were added to samples, as published previously (33). PCRs were run using three sets of specific primers, as follows: for the prokaryotic 16S rRNA gene, 515F/806R (34); for the eukaryotic 18S rRNA gene, F1427/R1616 (35); and for the fungal internally transcribed spacer, ITSF1/ITSF2 (36). The 16S rRNA primers are designed to amplify a broad range of archaeal and bacterial phyla, including cyanobacteria, and target plant organelle DNA as well; 18S rRNA primers amplify a broad range of eukaryotic taxa, protists, fungi, algae, diatoms, animals, and molds; and the ITS primers are designed for targeting soil fungal DNA, with a potential skew toward Ascomycota and Basidiomycota. The 18S rRNA primers differ from the ITS primers as they target a different part of the rRNA operon, have a wider target range, and have a more consistent amplicon size. However, the fungal 18S rRNA (or actually its part being amplified) is often not specific enough for genus-level phylogenetic analysis. Hence, we are using fungus-specific ITS primers that may not amplify the whole fungal community; however, they allow for a genus-level community assignment.

A two-step, double-indexing PCR system was used (37). The PCR mixture contents were as follows: Phusion high-fidelity, 0.2 μl; high-fidelity (HF) buffer, 4 μl (F520L; Thermo Scientific, Waltham, MA, USA); dinucleoside triphosphates (dNTPs), 0.4 μl; primers, 1 μl of 10 μM for each; template DNA, 1.5 μl of 5 ng/μl; and H_2_O to 20 μl. The PCR conditions were 98°C for 1 min, 35 cycles of 98°C for 15 s, 57°C for 15 s, and 72°C for 30 s, and a final elongation step of 72°C for 7 min. The second amplification was performed using dual-barcoded primers targeting the 12-bp pads flanking the DNA template from the first PCR round. The PCR conditions were as described above, with the exception that the cycle number was reduced to 25, and the annealing temperature was increased to 61°C. The final PCR products were pooled and purified as described above. Samples were sequenced using an Illumina MiSeq 300PE platform.

With the exception of the rhizoplane fraction of *A. thaliana* plants grown in Bawburgh soil using ITSF1/ITSF2 (fungal primers), PCR products were successfully obtained with the three pairs of primers from all fractions (covering all combinations of plants and soils).

For each primer pair (amplicon group), we ran water (“no DNA”) controls. These water controls had unsuccessful amplification during PCR as shown using agarose gel electrophoresis. However, there were sporadic “false-positive” outcomes probably when some DNA was split between wells of 96-well plates used throughout the PCR step (38).

### Processing sequence data.

Paired-end reads were aligned and binned according to the primer-binding and barcode sequences. Reads were merged using usearch10 fastq_mergepairs and quality filtered using fastq_maxee using EE at 1.0 (expected maximum number of errors per sequence). All reads longer or shorter than the expected amplicon size, at 292 bp ± 2 bp for bacteria, 210 to 220 bp for eukaryotes, and 200 to 300 bp for fungi (the ITS has a greater size variation) were also removed using a custom-made script. For further filtering, all reads were initially annotated using the curated SILVA (39), PR2 (40), and ITSoneDB (41) databases for prokaryotic 16S rRNA, eukaryotic 18S rRNA, and fungal ITS, respectively, and sequences of plant origin (chloroplast, mitochondrion, and genomic chromosomes) were removed. We used 3,000 reads per sample (1,000 for each amplicon group) following plant/organelle DNA removal. Most of the eukaryotic samples (based on the 18S rRNA sequencing) had a high content of plant DNA (up to ∼80%). After removal of these reads, for consistency, we reduced every sample to 1,000 reads. For analysis resulting in Venn diagrams, Shannon diversity index bar charts, similarity percentage (SIMPER) analysis, permutational multivariate analysis of variance (PERMANOVA), and unconstrained principal-coordinate analysis (PCoA) plots, reads were binned into zero-radius operational taxonomic units (zOTUs), including chimera removal according to the Usearch10 pipeline with UNOISE3 (an update from Unoise2) (42). Reads forming zOTUs were annotated using the same databases set as described above. For the complete, annotated script used for data analysis, please refer to Table S1, sheet 7. We also performed some tests using slightly different sequencing filtering methods, OTU clustering (cluster_fast using different identification [ID] thresholds), and visualization methods (such as nonmetric multidimensional scaling [NMDS]) (data not shown). Regardless of the methods used, the resulting main data pattern observed was always the same.

### Statistical analysis.

PCoA was performed using standardized, square-root-transformed data and analyzed using the Bray-Curtis coefficient. PERMANOVA was used to detect significant changes in the microbiota structure using pseudo-F value as a proxy for the strength of an individual factor (fraction, soil, or plant species) on the community composition. A high pseudo-F value indicates that the difference (variability) between groups of samples is higher than the variability inside these groups. The *P* value as a measure of significance of the pseudo-F values was determined using 9,999 permutations (analysis restarts using random sample group allocation). All of the PERMANOVA output is provided in Table S1, sheet 1. The taxa with higher contributions to community dissimilarity were identified using SIMPER. The PRIMER 6 software (PRIMER-E, Plymouth, UK) was used for PCoA construction as well as PERMANOVA and SIMPER calculations (43). For Venn diagrams, we are only analyzing zOTUs found in at least 50% of the biological replicates, hence reducing their overall numbers; i.e., when 184 zOTU are shown to be shared between unplanted soils, it means that 184 zOTU were both found in at least 50% of replicates of Bawburgh soil and in at least 50% replicates of Wytham soil samples. Data were visualized using the Microsoft Excel, GraphPad Prism, and OmniGraffle software programs.

### Data availability.

The sequences generated for this study are deposited in the European Nucleotide Archive (ENA) under the accession number PRJEB27065.
