# Supplementary figures and images for: Influence of Plant Fraction, Soil, and Plant Species on Microbiota: a Multikingdom Comparison
Source: mBio. 2020 Feb 4;11(1):e02785-19. doi: 10.1128/mBio.02785-19 (PMC7002342; doi:10.1128/mBio.02785-19)

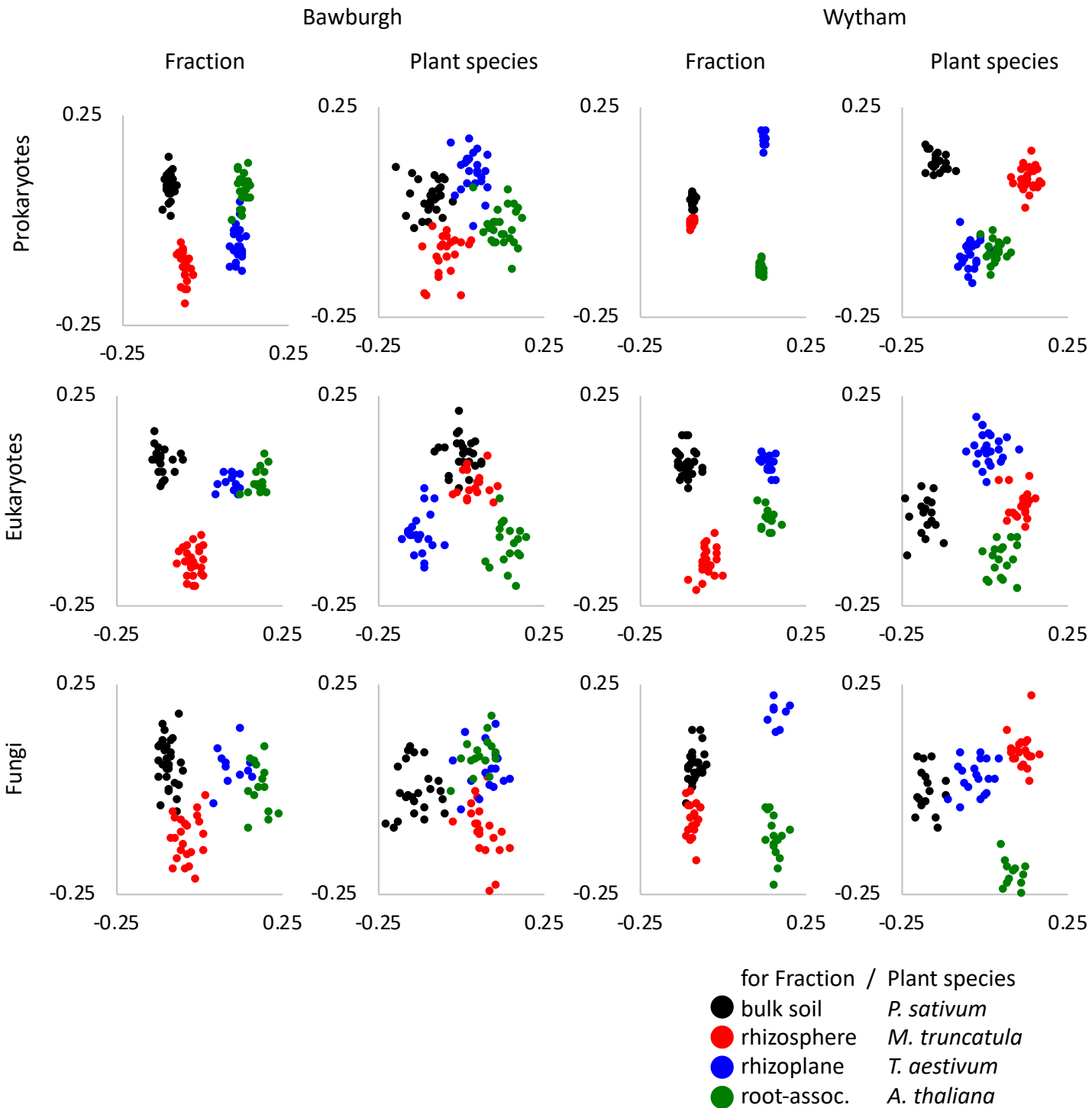

Supplement: FIG S1 [file mBio.02785-19-sf001.pdf]

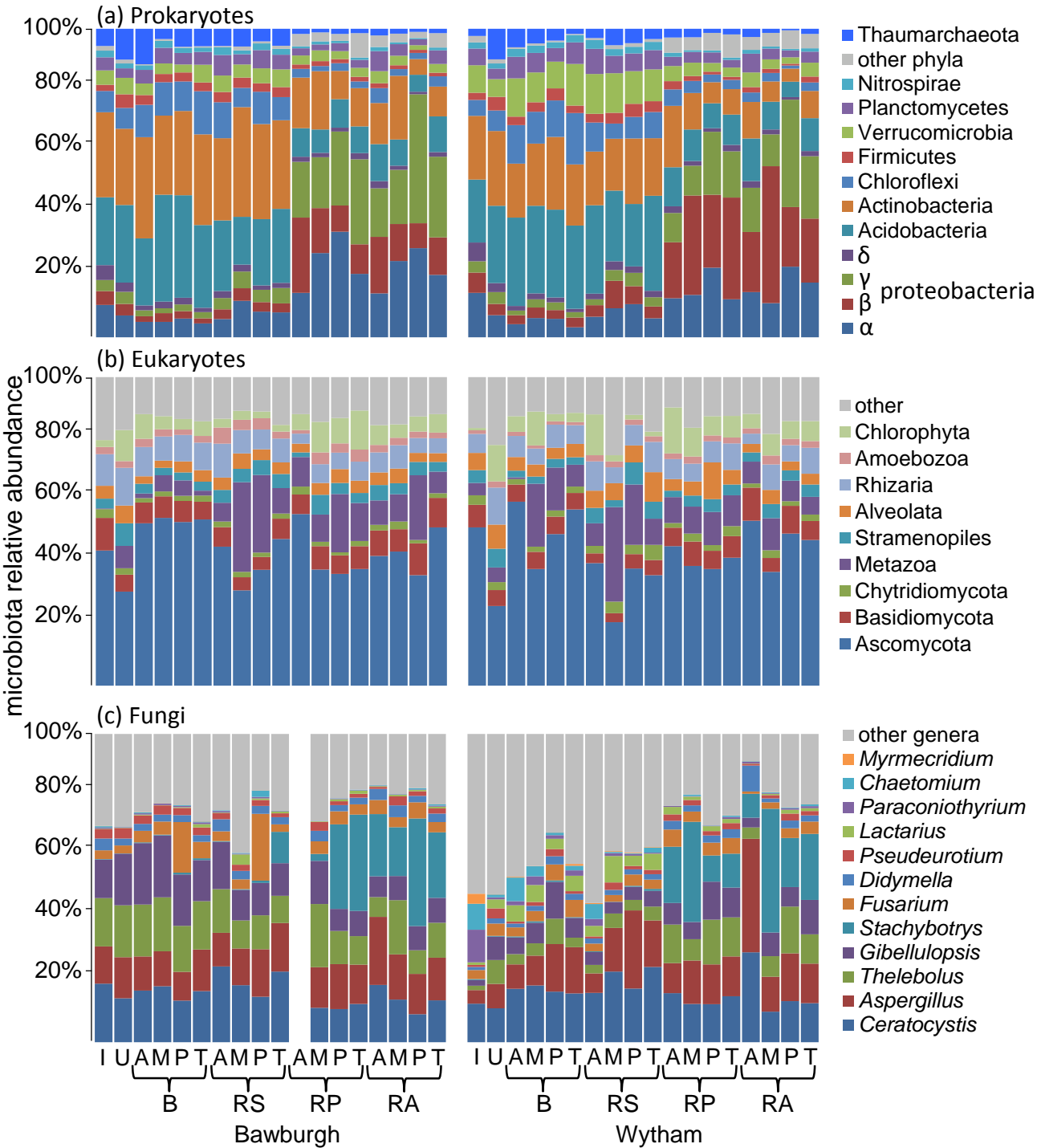

Supplement: FIG S2 [file mBio.02785-19-sf002.pdf]

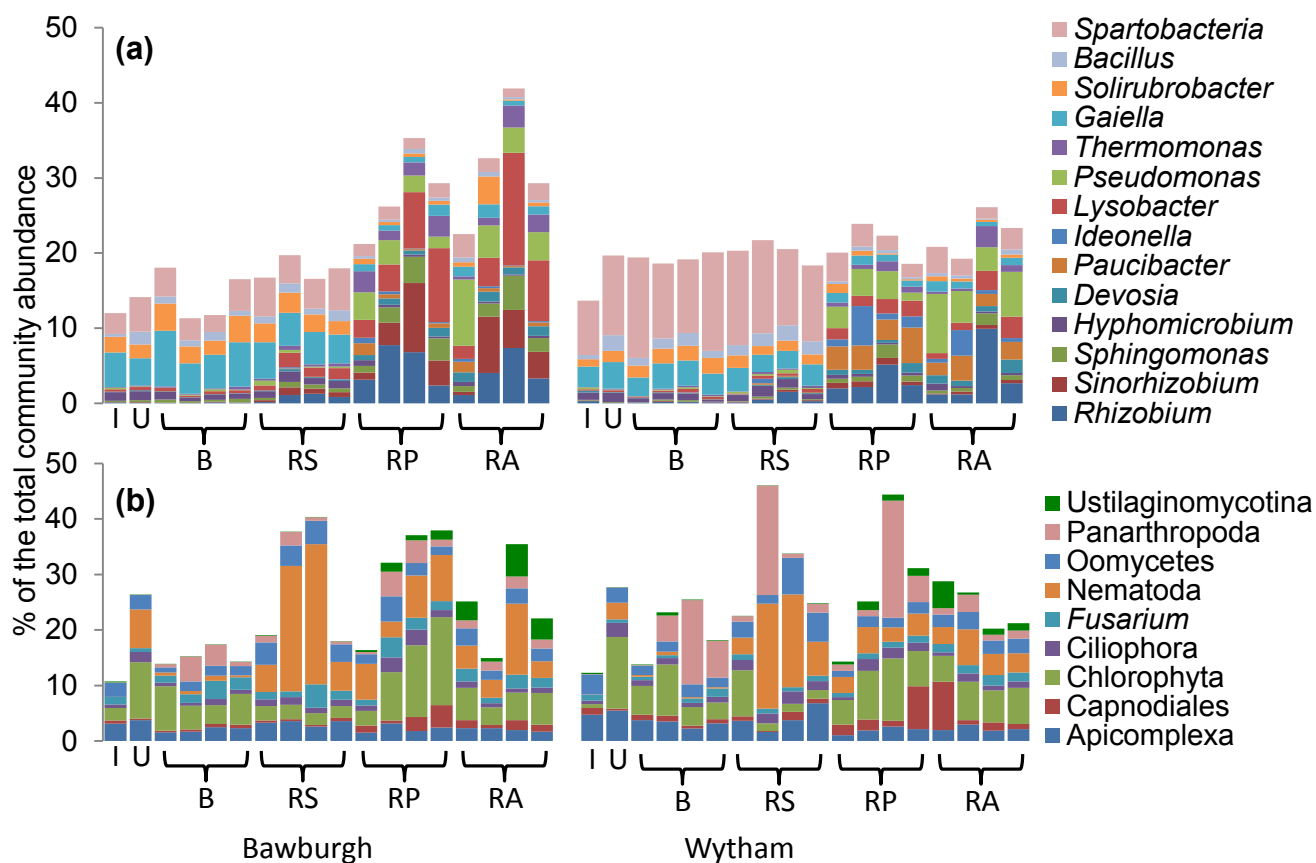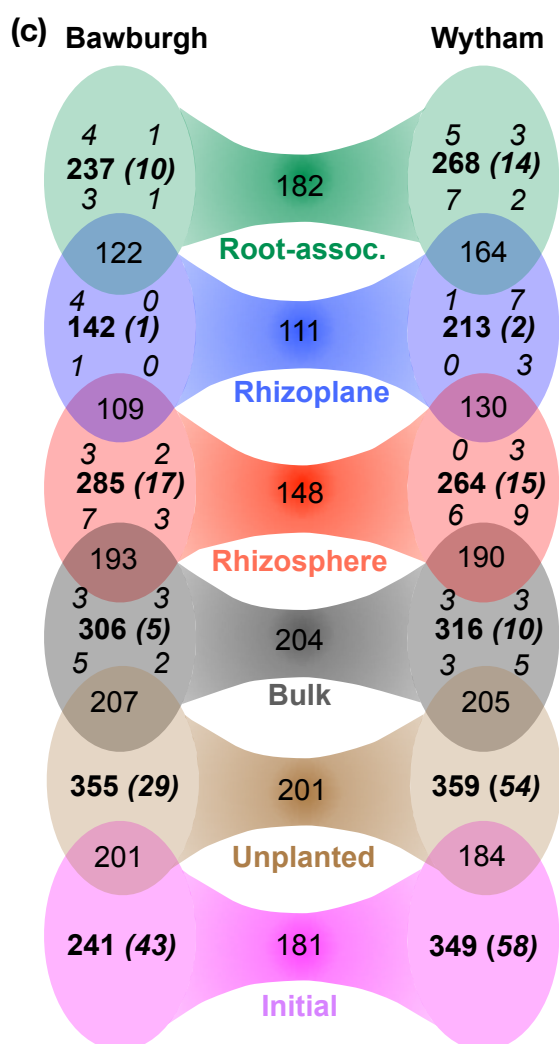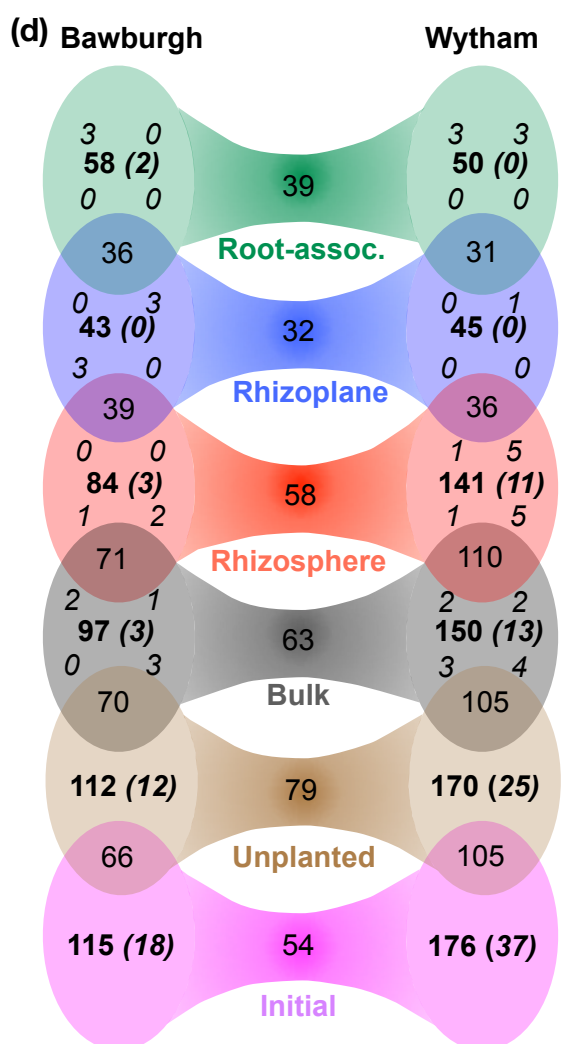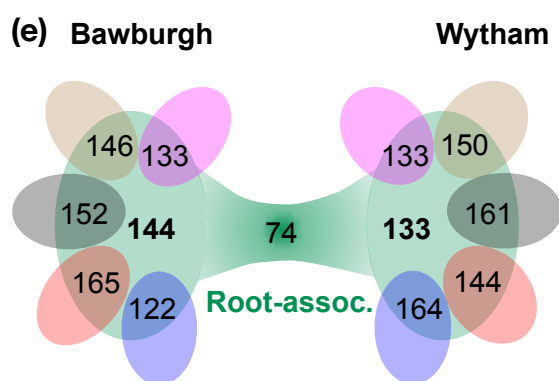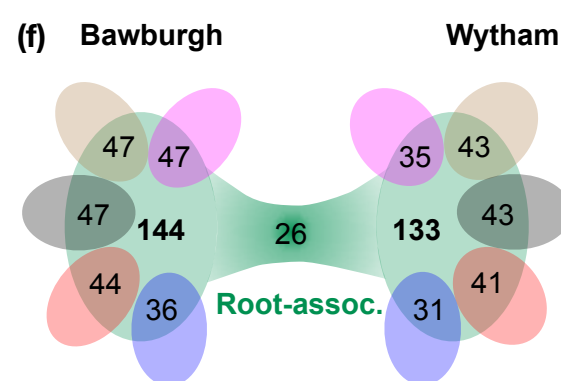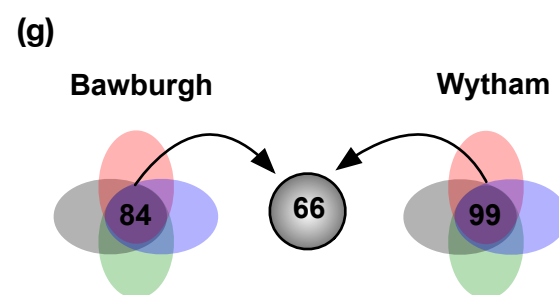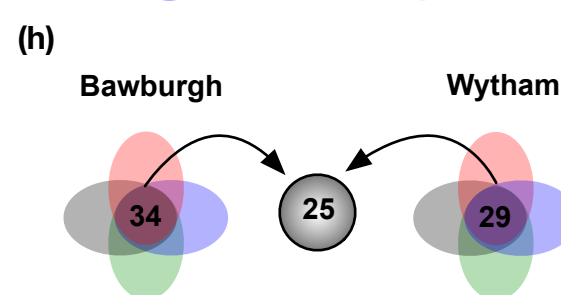

Supplement: FIG S3 [file mBio.02785-19-sf003.pdf]

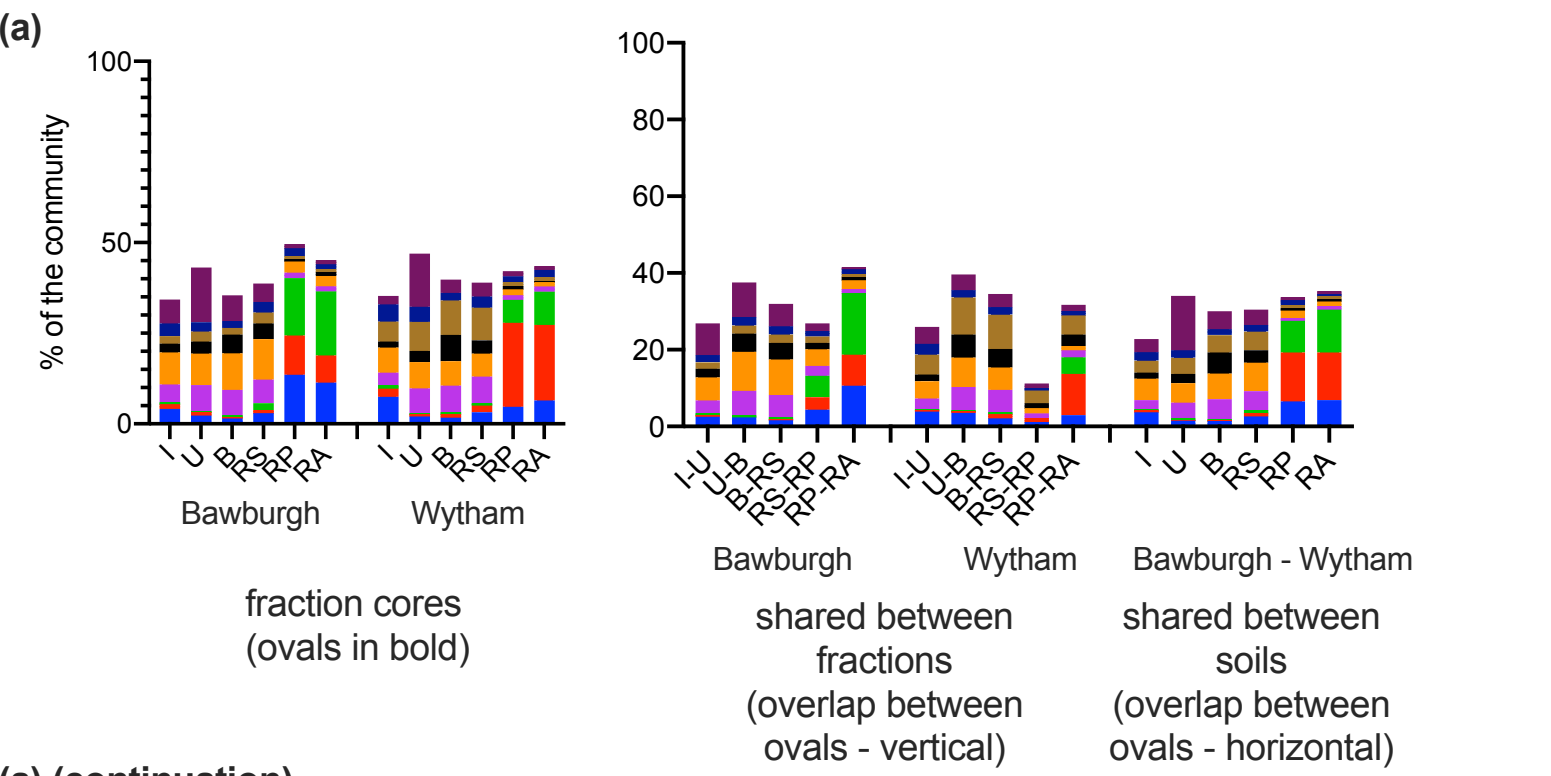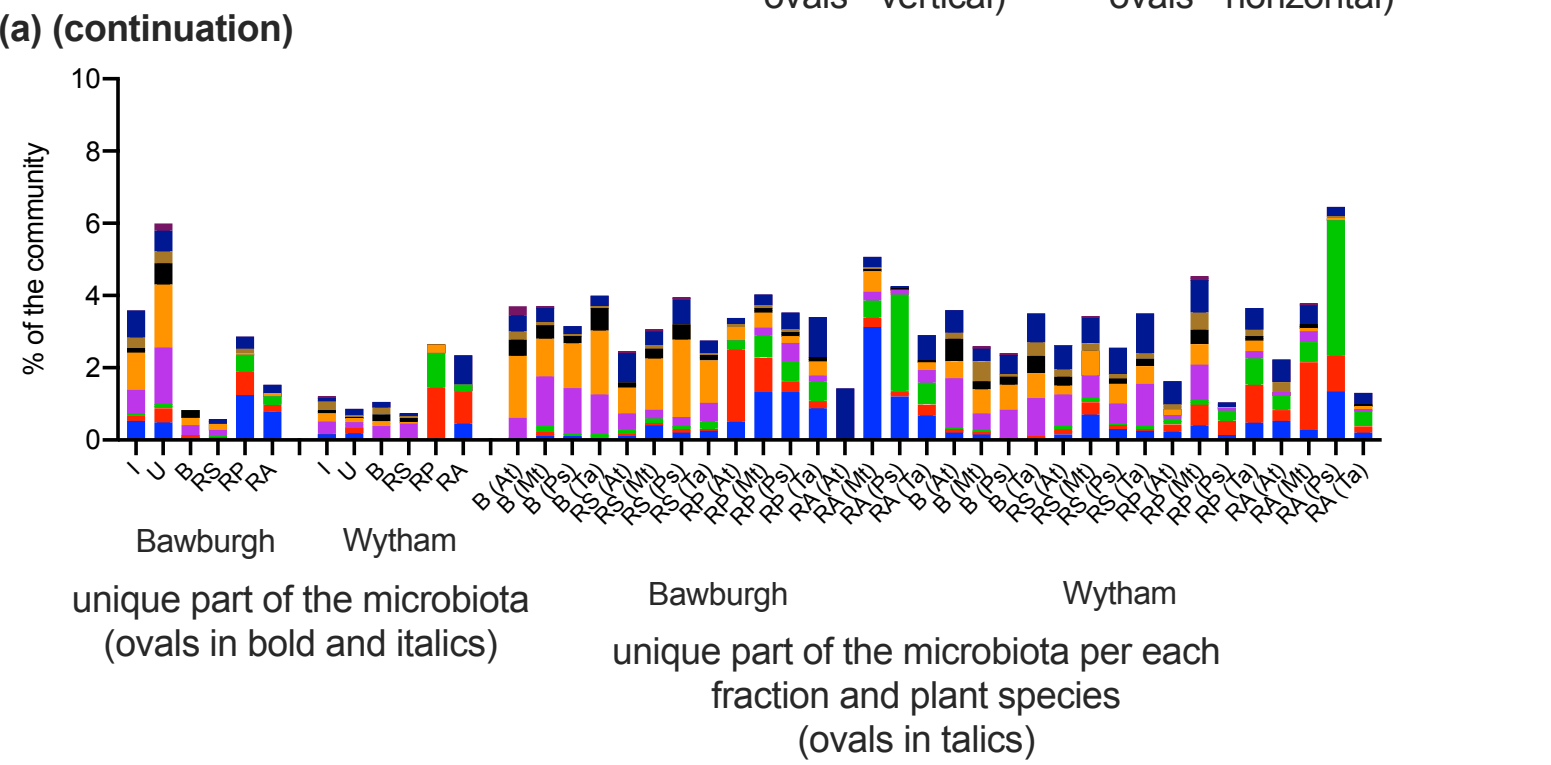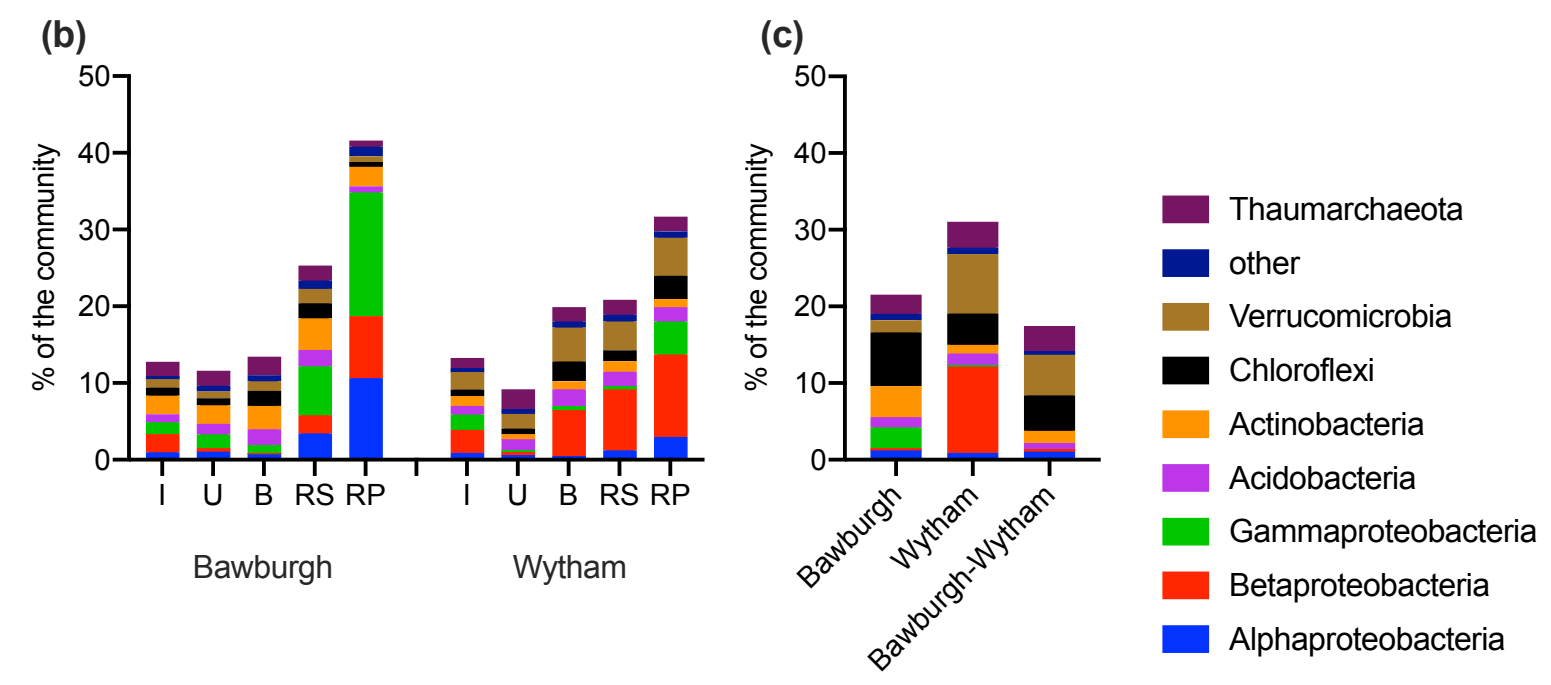

Supplement: FIG S5 [file mBio.02785-19-sf005.pdf]

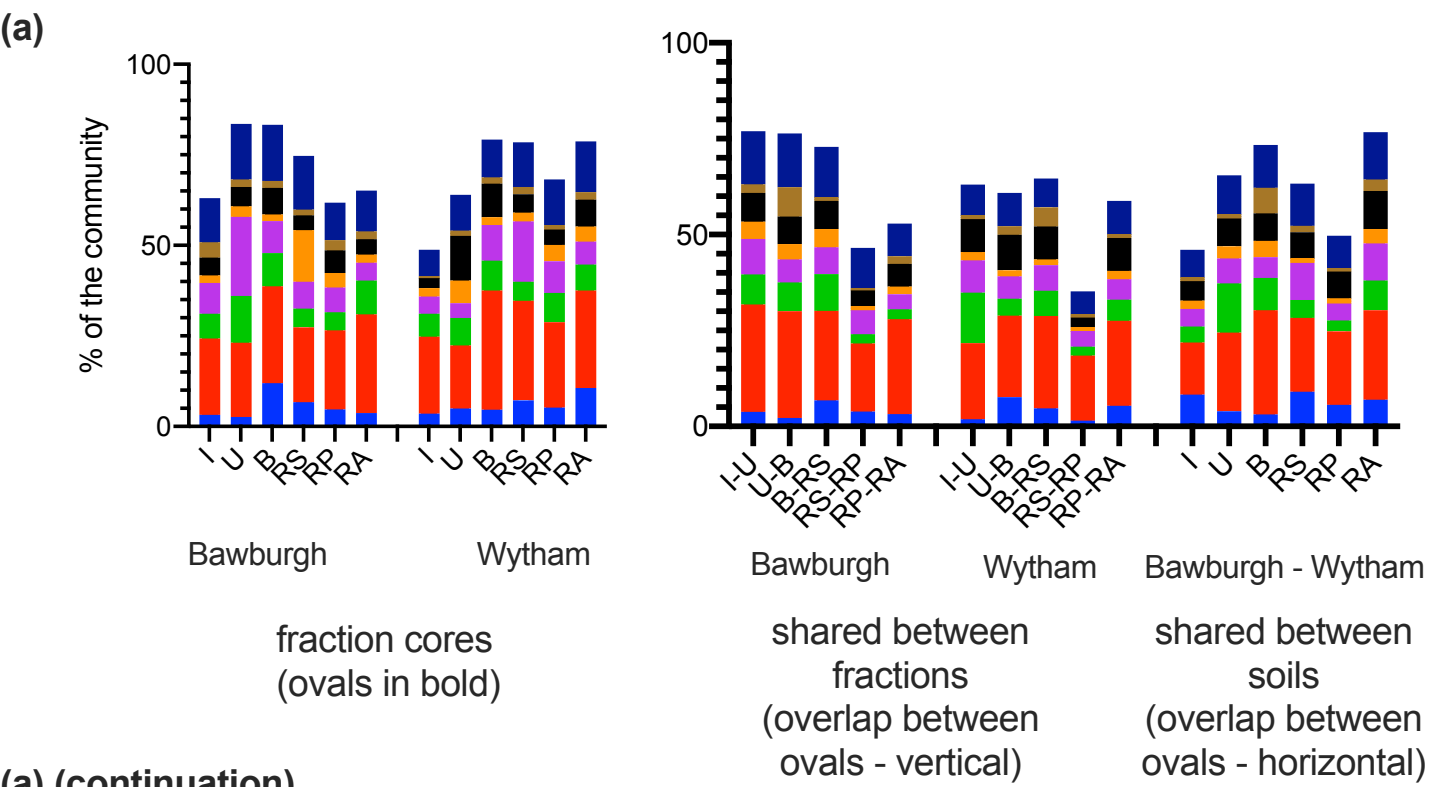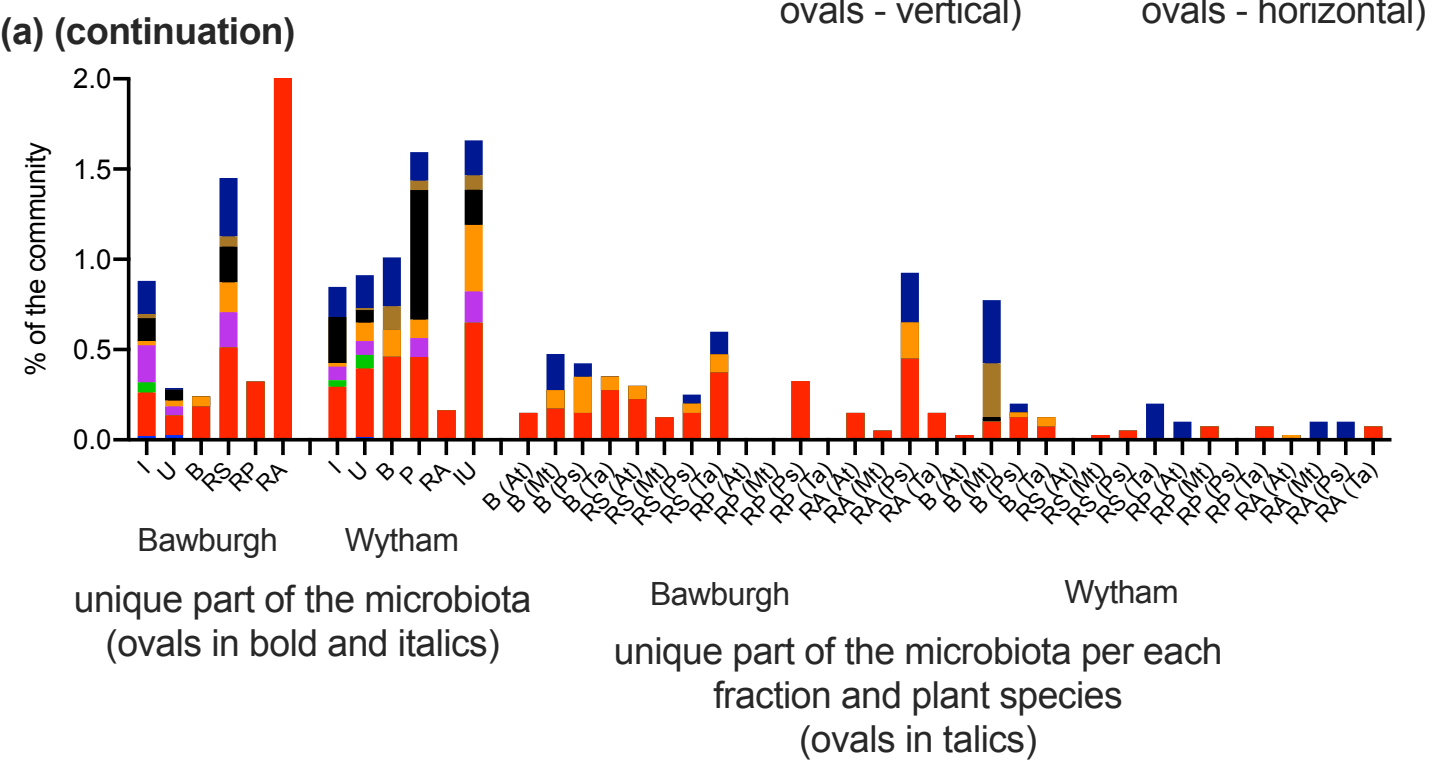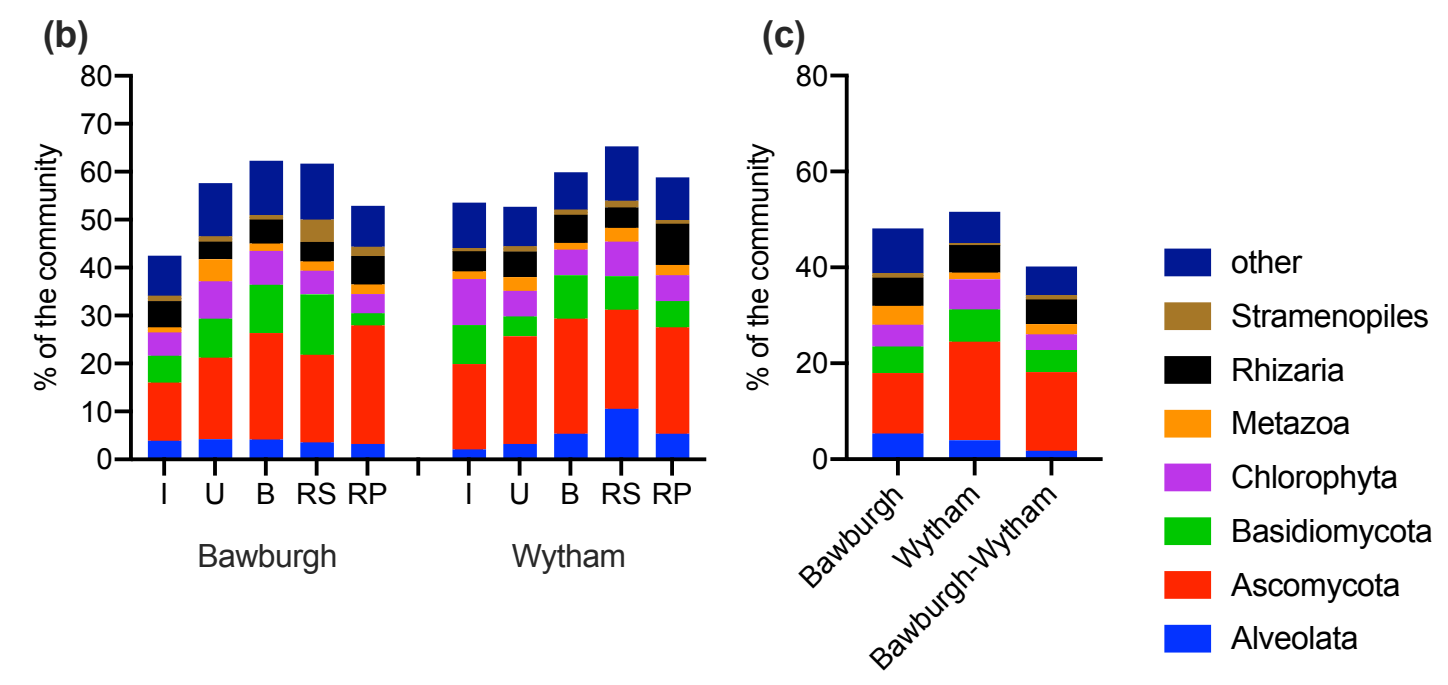

Supplement: FIG S6 [file mBio.02785-19-sf006.pdf]

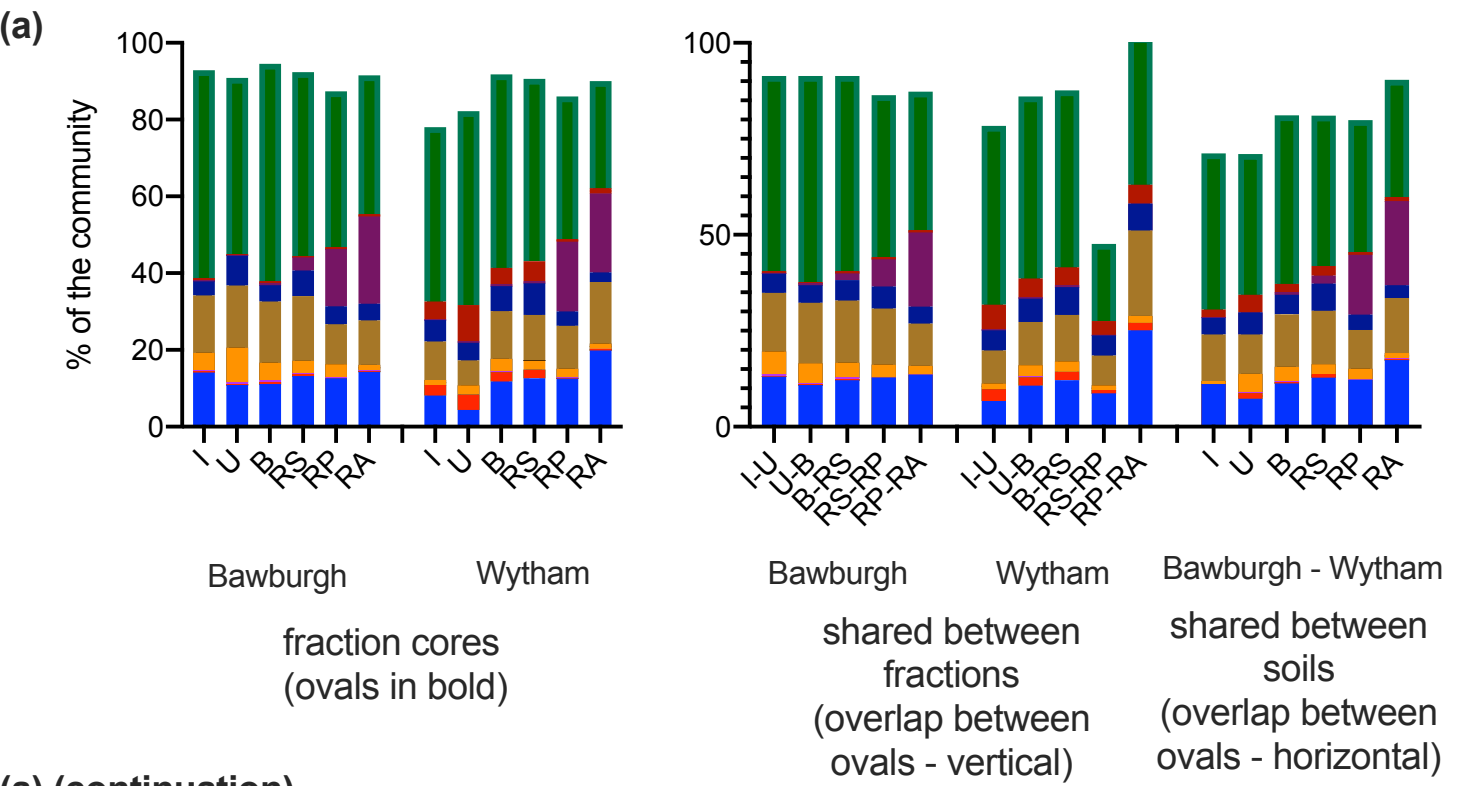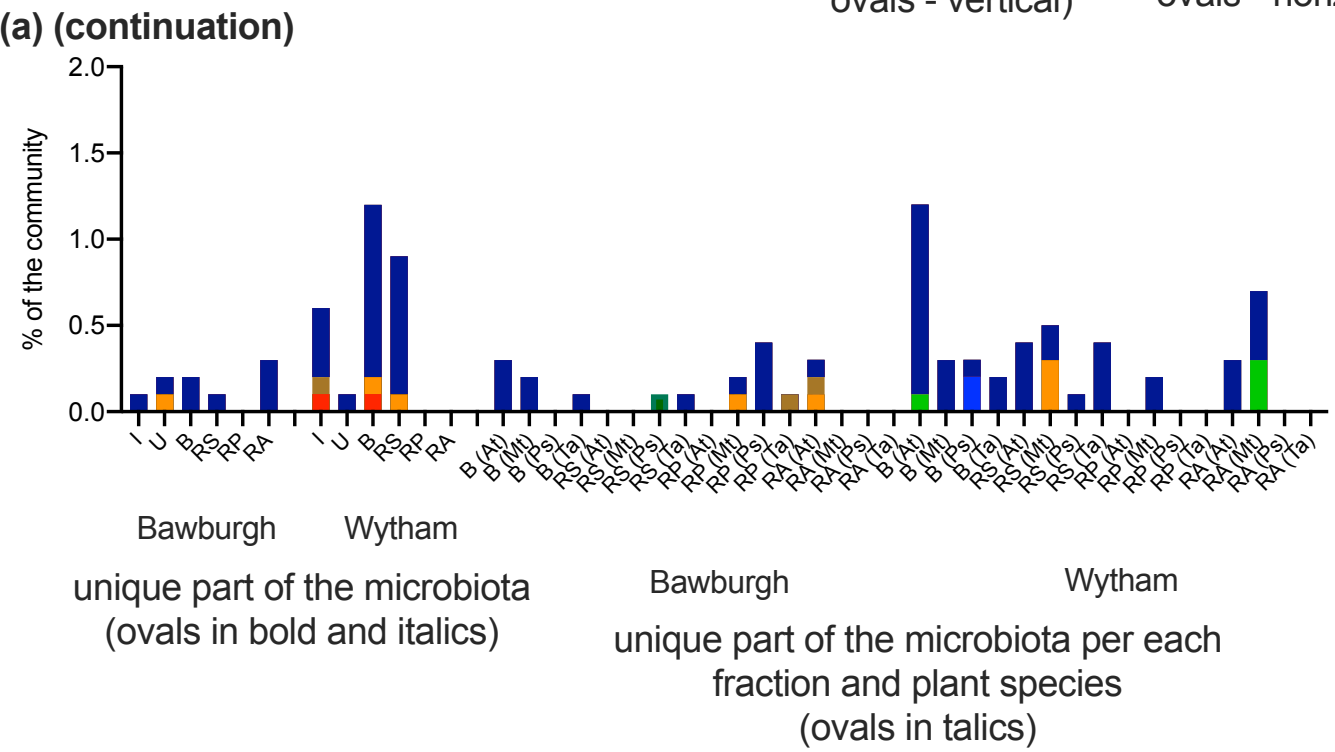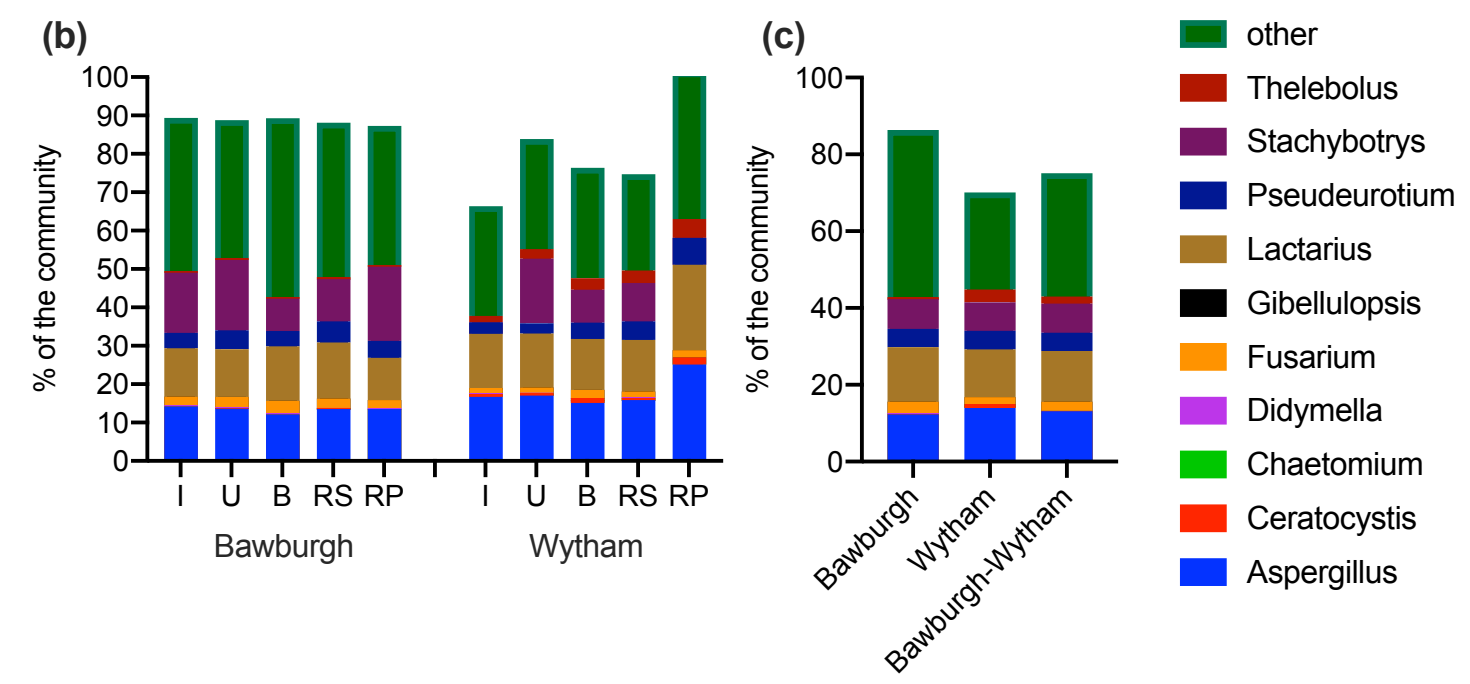

Supplement: FIG S7 [file mBio.02785-19-sf007.pdf]

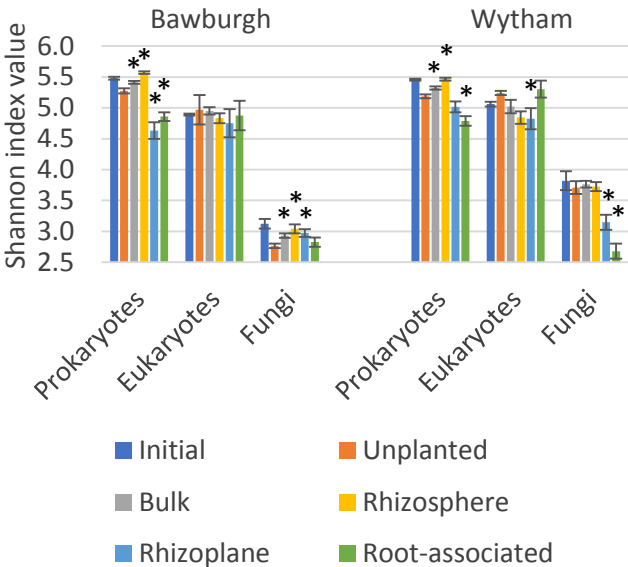

Supplement: FIG S8 [file mBio.02785-19-sf008.pdf]

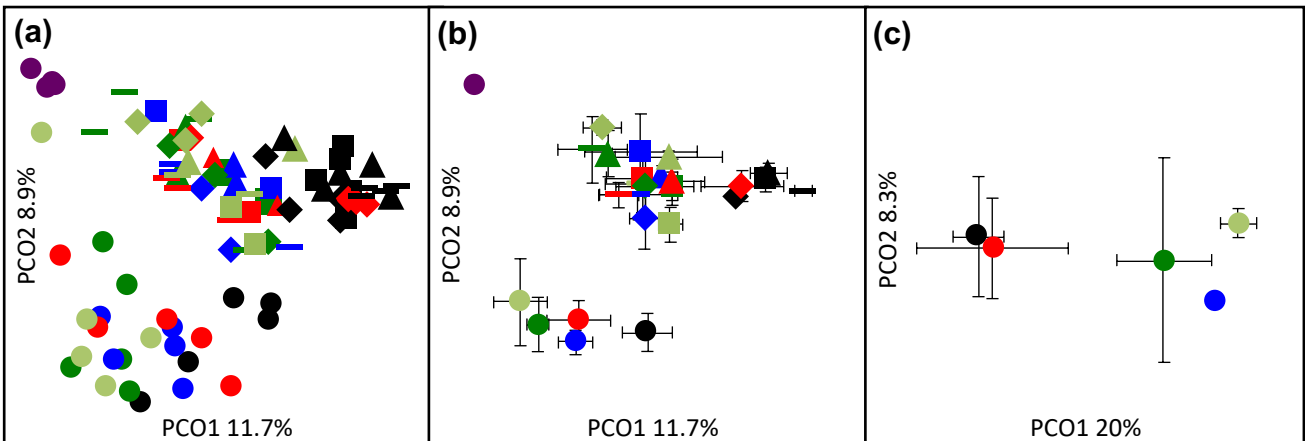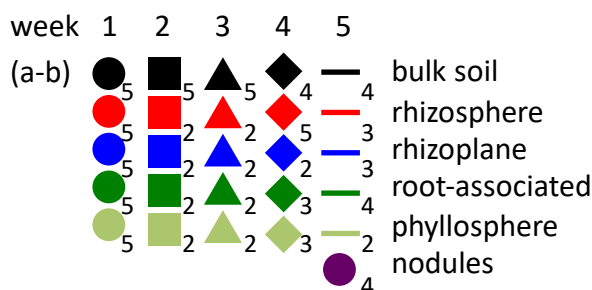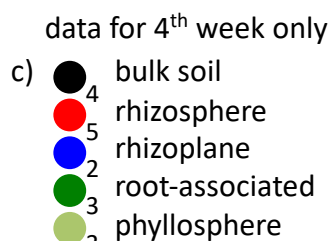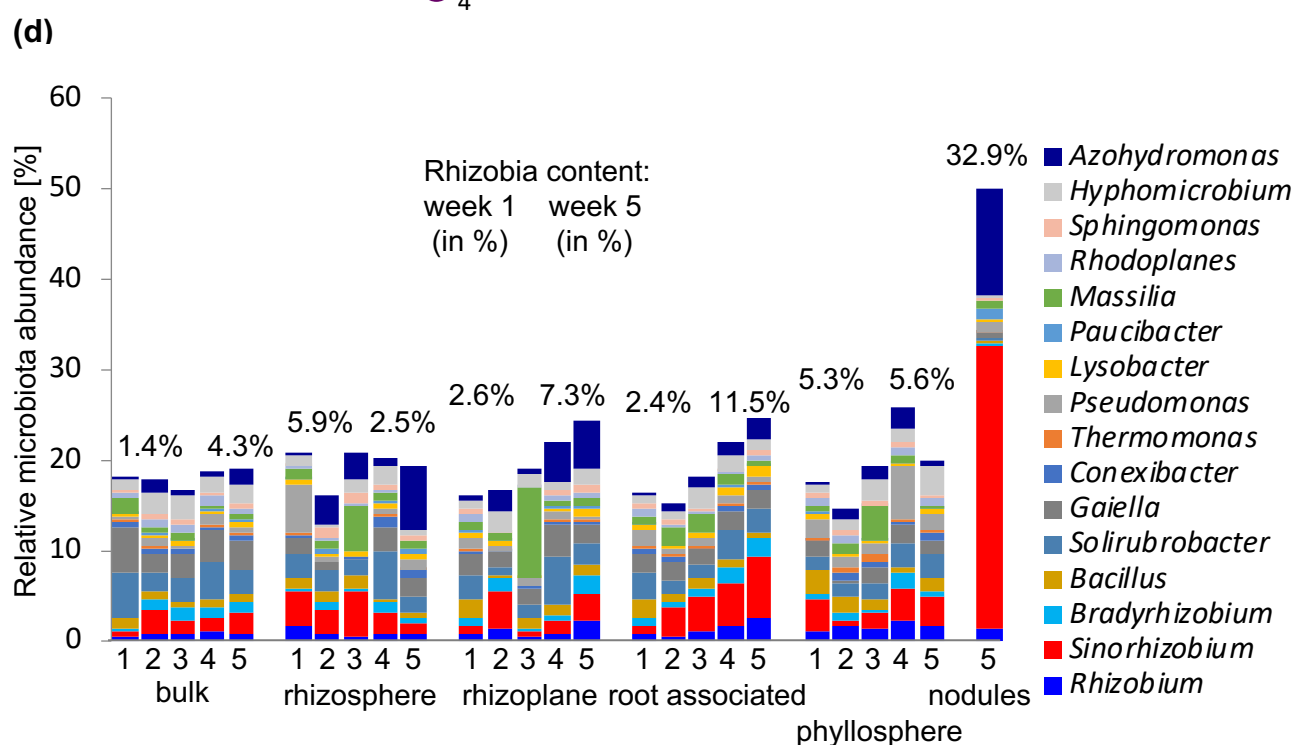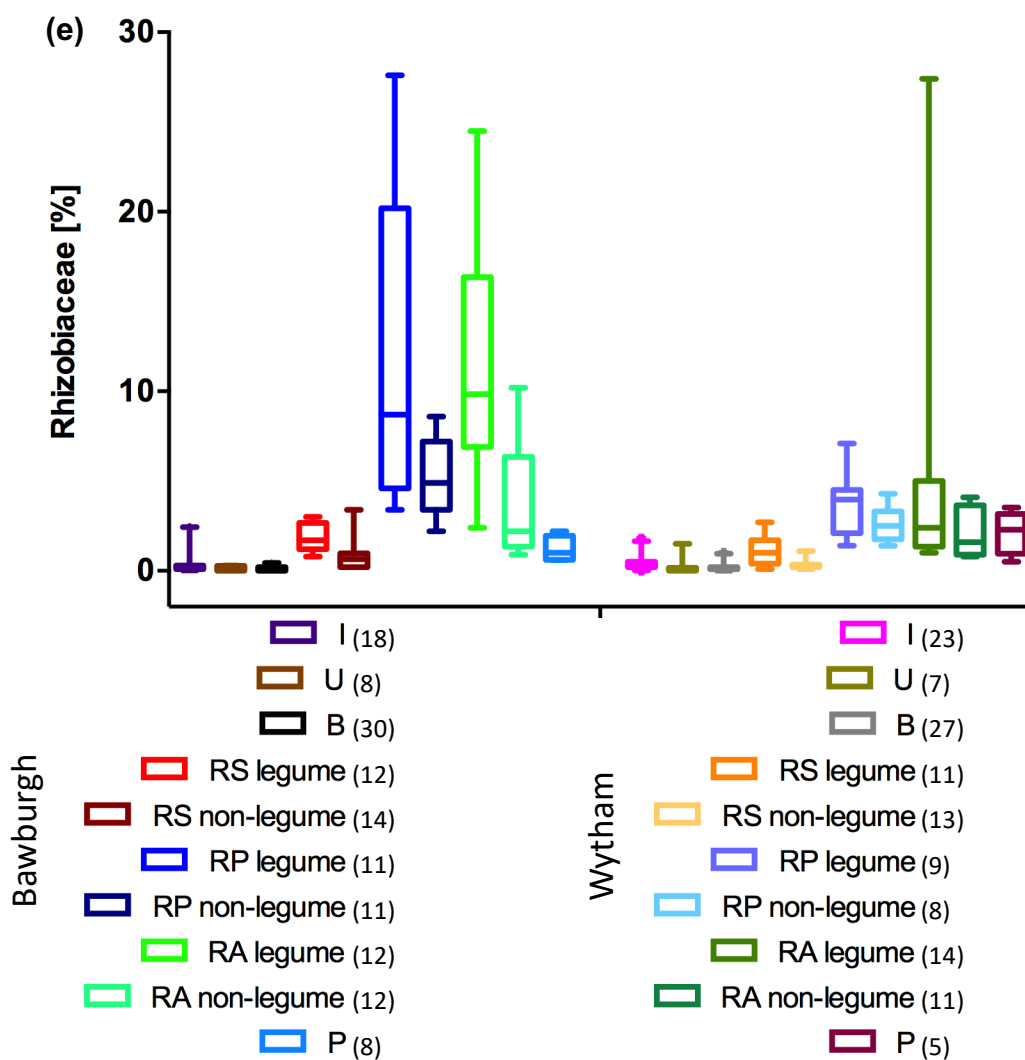

Supplement: FIG S9 [file mBio.02785-19-sf009.pdf]
