# Supplementary material for: Influence of Plant Fraction, Soil, and Plant Species on Microbiota: a Multikingdom Comparison
Source: mBio. 2020 Feb 4;11(1):e02785-19. doi: 10.1128/mBio.02785-19 (PMC7002342; doi:10.1128/mBio.02785-19)
Supplement: FIG S4 [file mBio.02785-19-sf004.pdf]

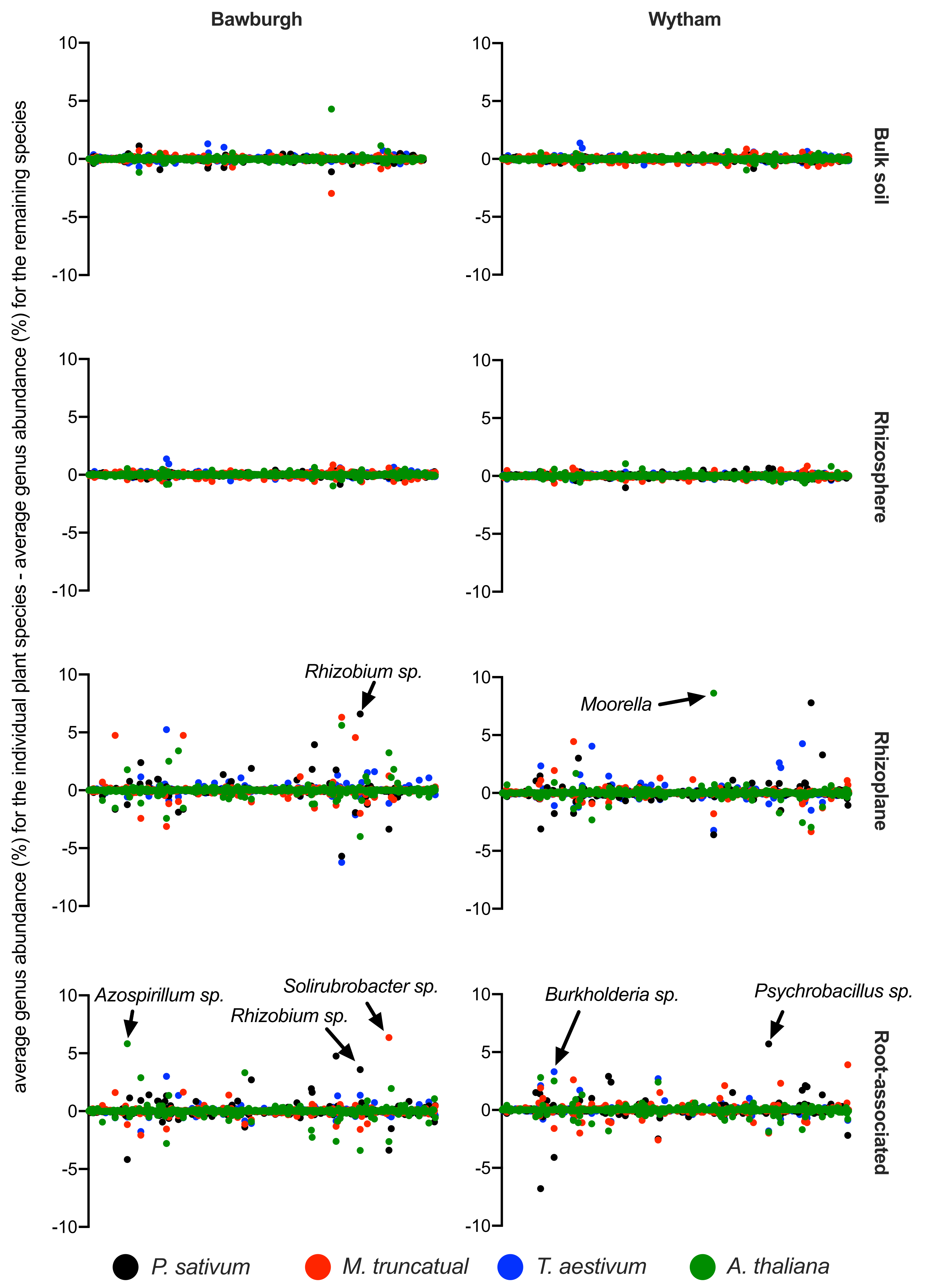

Bawburgh
Wytham
Bulk soil
Rhizosphere
Rhizoplane
Root-associated
average genus abundance (%) for the individual plant species - average genus abundance (%) for the remaining species
Rhizobium sp.
Moorella
Azospirillum sp.
Solirubrobacter sp.
Rhizobium sp.
Burkholderia sp.
Psychrobacillus sp.
10
5
0
-5
-10
P. sativum
M. truncatual
T. aestivum
A. thaliana
